# Warfare among rice sheath pathogens: *Rhizoctonia solani* AG 1-IA neutralizes *Pseudomonas fuscovaginae* cyclic lipopeptides

**DOI:** 10.1128/aem.01524-25

**Published:** 2026-01-16

**Authors:** Jasmine De Rop, Durga Prasad, Niels Geudens, Lu Zhou, Pieter Spanoghe, José C. Martins, Monica Höfte

**Affiliations:** 1 Laboratory of Crop Protection Chemistry, Department of Plants and Crops, Faculty of Bioscience Engineering, Ghent University 26656, Ghent, Belgium; 2 Laboratory of Phytopathology, Department of Plants and Crops, Faculty of Bioscience Engineering, Ghent University 26656, Ghent, Belgium; 3 NMR and Structure Analysis Unit, Department of Organic and Macromolecular Chemistry, Faculty of Sciences, Ghent University 26656, Ghent, Belgium; Michigan State University, East Lansing, Michigan, USA

**Keywords:** rice sheath brown rot, fuscopeptin, syringotoxin, cyclic lipopeptides, rhizoctonia

## Abstract

In this work, we investigated the chemical process underlying the interplay between two rice sheath pathogens: *Pseudomonas fuscovaginae*, the causal agent of sheath brown rot, and *Rhizoctonia solani* AG 1-IA, which causes sheath blight. Specifically, we studied the fate of the bacterial cyclic lipopeptides (CLiPs) syringotoxin and fuscopeptin in this interaction. Both compounds exhibit potent antifungal activity against *R. solani* and induce phytotoxic effects. Ultra-performance liquid chromatography-tandem mass spectrometry analysis demonstrated that *R. solani* AG 1-IA likely secretes two distinct enzymes: an esterase that hydrolyzes the ester bond in syringotoxin, producing a linear derivative, and a protease that cleaves the glycine-alanine bond within the peptide backbone of fuscopeptin. The degradation products lack antifungal and phytotoxic activities, nullifying the competitive advantage of *P. fuscovaginae*. These enzymatic effects showed increased activity at 28°C. In contrast, *R. solani* AG 2-2 did not degrade CLiPs. Further analysis with a broader range of *R. solani* isolates revealed that CLiP degradation is a common trait among AG 1-IA isolates. This study provides the first evidence that *R. solani* AG 1-IA actively neutralizes the antifungal and phytotoxic activities of *P. fuscovaginae* through targeted enzymatic degradation.

**IMPORTANCE:**

Rice is a global staple crop that is susceptible to various pathogens, including *Pseudomonas fuscovaginae*, causing sheath brown rot, and *Rhizoctonia solani* AG 1-IA, which causes sheath blight. Notably, *P. fuscovaginae* primarily occurs at lower temperatures, whereas *R. solani* AG 1-IA is more prevalent under warmer conditions. Previous research demonstrated that *P. fuscovaginae* produces higher levels of the virulence-associated cyclic lipopeptides (CLiPs) syringotoxin and fuscopeptin at 18°C, potentially explaining its pathogenicity on rice plants grown at high altitudes. Conversely, *R. solani* AG 1-IA, which is sensitive to these CLiPs, was found to secrete CLiP-degrading enzymes, with degradation activity enhanced at 28°C. When combined with the reduced CLiP production by *P. fuscovaginae* at higher temperatures, this enzymatic degradation may confer a competitive advantage to *R. solani* in warmer environments. The absence of reports documenting the co-occurrence of both pathogens in field conditions may, at least in part, be explained by this temperature-dependent antagonism.

## INTRODUCTION

*Rhizoctonia solani* is a soilborne fungal pathogen responsible for a wide range of plant diseases, including damping-off, hypocotyl rot, and foliar infections. It is grouped into 14 anastomosis groups (AG 1-13 and BI) based on genetic relatedness, hyphal anastomosis behavior, cultural morphology, host range, and pathogenicity (1, 2). Among them, *R. solani* AG 1-IA is the primary causal agent of rice sheath blight, a severe disease in intensive rice production systems (3, 4). This pathogen thrives at temperatures between 25°C and 30°C, with high humidity playing a critical role in its survival and aggressiveness (5–7). *R. solani* AG 2-2 is a soilborne pathogen that causes root and hypocotyl rot on a range of hosts, including cauliflower, endive, maize, and bean, leading to severe yield losses (8–13).

Another notable rice pathogen is *Pseudomonas fuscovaginae* UPB0736, a member of the *Pseudomonas asplenii* subgroup within the *Pseudomonas fluorescens* group that causes bacterial sheath brown rot. This disease leads to significant agricultural damage through grain discoloration, poor spike emergence, and panicle sterility. Additionally, *P. fuscovaginae* has been reported to infect wheat, maize, and sorghum, causing brown rot lesions on leaf sheaths and husks, particularly in high-altitude regions (14–18). Unlike *R. solani* AG 1-IA, *P. fuscovaginae* predominantly causes disease in cooler, high-altitude rice-growing environments where temperatures typically range between 18°C and 23°C or lower (15, 17, 19–21).

*Pseudomonas* species are well known for their ability to synthesize cyclic lipopeptides (CLiPs), which are amphiphilic specialized metabolites with diverse biological functions, including antimicrobial and antifungal activity, phytotoxicity, induced systemic resistance in plants, and roles in motility, biofilm formation, and root colonization (22–25). Specifically, *P. fuscovaginae* UPB0736 produces three well-characterized CLiPs: syringotoxin, fuscopeptin, and asplenin (26). Syringotoxin, classified within the Mycin family, features a 9-amino-acid macrolactone ring and a 3-hydroxy fatty acid tail comprising 14 carbons (C14:0). It exhibits strong antifungal activity against *R. solani* due to its ability to form pores in biological membranes and also contributes to sheath brown rot disease pathogenesis (26–30). Fuscopeptins, belonging to the Peptin family, consist of two structural variants—fuscopeptin A and fuscopeptin B—distinguished by their C8:0 and C10:0 acyl chains, respectively. These CLiPs exhibit phytotoxicity by inhibiting plant H^+^-ATPases and permeabilizing membranes, thereby disrupting host cellular processes (31, 32), and are important virulence factors in rice (26). Additionally, *P. fuscovaginae* UPB0736 produces asplenin, a CLiP characterized by a 13-amino-acid sequence with an 8-amino-acid macrolactone ring. Unlike syringotoxin and fuscopeptin, asplenin does not exhibit antifungal or phytotoxic activity but plays a key role in bacterial motility (26).

Notably, *P. fuscovaginae* UPB0736 is known to inhibit the growth of *R. solani* and other fungal pathogens mainly through the antifungal action of syringotoxin (26, 33). We previously showed that the optimal temperature for CLiP production by *P. fuscovaginae* UPB0736 is 18°C. In addition, *R. solani* AG 1-IA is more sensitive to syringotoxin and fuscopeptin at 18°C (IC_50_ of 0.02 mg/L for syringotoxin and 2.66 mg/L for fuscopeptin) than at 28°C (IC_50_ of 0.11 mg/L for syringotoxin and 4.13 mg/L for fuscopeptin), while this is not the case for *R. solani* AG 2-2 (IC_50_ of 0.08 mg/L at both temperatures for syringotoxin and 9.68 mg/L at 18°C and 6.97 mg/L at 28°C for fuscopeptin) (34). This suggests that *R. solani* AG 1-IA is either more sensitive to syringotoxin and fuscopeptin at 18°C or more efficient at degrading these CLiPs at 28°C.

Although both *P. fuscovaginae* UPB0736 and *R. solani* AG 1-IA target the rice sheath, their co-occurrence under field conditions has, to our knowledge, not been reported. We hypothesize that temperature-dependent antagonism underlies this mutual exclusivity: at 18°C, elevated CLiP production enhances bacterial virulence and suppresses fungal growth, while at 28°C, reduced CLiP production combined with fungal enzymatic degradation shifts the competitive balance in favor of *R. solani* AG 1-IA.

## MATERIALS AND METHODS

### Strains and culture conditions

The strains used in this study are listed in Table 1. All *Pseudomonas* strains were cultured in King’s B liquid medium (KB; 10 g/L proteose peptone No. 3 [Difco], 1.5 g/L K_2_HPO_4_, 2.96 g/L MgSO_4_ × 7 H_2_O, and 10 mL/L glycerol) or potato dextrose broth (PDB, Becton Dickinson) at 18°C. Solidified KB medium was prepared by supplementing it with 15 g/L of bacteriological agar (Difco). Liquid cultures were incubated in baffled flasks with a 15% filling volume and shaken at 150 rpm. *Rhizoctonia* strains (Table 1) were cultured on potato dextrose agar (PDA; Becton Dickinson) at 28°C, while liquid cultures were grown in PDB at either 18°C or 28°C under the same shaking conditions (150 rpm in baffled flasks with a 15% filling volume).

**TABLE 1:** Strains used in this study and their main characteristics

Strains	Origin and characteristics	Reference
Bacteria^*a*^		
*P. fuscovaginae*		
UPB0736 WT	Wild type (*Oryza sativa*, Madagascar)	26
UPB0736 Δ*fst* Δ*fus*	Deletion in *fstA* and *fusA* genes	26
UPB0736 Δ*fst*	Deletion in *fstA* gene	26
UPB0736 Δ*fus*	Deletion in *fusA* gene	26
UPB0736 Δ*fst* Δ*asp*	Deletion in *fstA* and *aspA* genes	26
UPB0736 Δ*fus* Δ*asp*	Deletion in *fusA* and *aspA* genes	26
Fungi		
*R. solani* AG 2-2 CuHav-Rs18	*Phaseolus vulgaris*, Cuba	10
*R. solani* AG 1-IA STMX04-03	Cabbage, Vietnam, pathogenic on rice	35
*R. solani* AG 1-IA STMX04-4	Chinese cabbage, Vietnam	35
*R. solani* AG 1-IA HNGL01-3	Vietnam	35
*R. solani* AG 1-IA STMX01-1	Pak Choi, Vietnam	35
*R. solani* AG 1-IA HNDD01-3	Turnip cabbage, Vietnam	35
*R. solani* AG 1-IA CTCR01-3	White cabbage, Vietnam	35
*R. solani* AG 1-IB LDDL05-3	White cabbage, Vietnam	35
*R. solani* AG 1-ID DNBH05-4	Chinese cabbage, Vietnam	35
*R. solani* AG 1-IG DNBH05-1-2	Chinese cabbage, Vietnam	35
*R. solani* AG 2-2 HNDA01-1	Turnip cabbage, Vietnam	35
*R. solani* AG 4-HGI STST01-1	Vietnam	35
*R. solani* AG 7 HNDA02-1	Turnip cabbage, Vietnam	35
*R. solani* AG A DNBH04-1	Mustard cabbage, Vietnam	35
*R. solani* AG Fc/V LDDL02-1	Chinese cabbage, Vietnam	35, 36

^*a*^ fst, syringotoxin; fus: fuscopeptin; asp, asplenin.

### *Pseudomonas* spp. metabolite extraction from liquid broth

For the metabolite extraction, 150 mL of the inoculated broth was centrifuged, and the cell-free supernatant (SN) was collected. Extraction of the CLiPs was done by solid-liquid extraction using Oasis HLB extraction columns (Waters, Milford, USA) (34). Columns were activated with 5 mL of methanol and 5 mL of MilliQ. Prior to elution, the column was washed with 5 mL 20% acetonitrile (ACN). The CLiPs were eluted with 5 mL 50% ACN, and the samples were analyzed with ultra-performance liquid chromatography-tandem mass spectrometry (UPLC-MS/MS, Waters, Milford, USA). The recovery of this method for syringotoxin is 96.0% ± 7.2%, and the recoveries of fuscopeptin A and B are 75.10% ± 6.1% and 78.12% ± 4.1%, respectively (*n* = 6). Due to the unavailability of sufficient purified asplenin standard, absolute quantification was not feasible. Consequently, asplenin levels were not reported.

### Chemical analysis of CLiPs produced by *Pseudomonas* spp.

The CLiPs were identified using UPLC-MS/MS performed on a triple quadrupole system with electrospray ionization (ESI) (Waters ACQUITY UPLC, Xevo TQD mass spectrometer) with the parameter set up as follows: capillary voltage of 2.5 kV, source temperature 130°C, desolvation temperature 450°C, desolvation gas flow 650 L/h, and cone gas flow 50 L/h. The ionization was kept in positive mode (ES+). Mass spectra were recorded for m/z ratios ranging from 100 to 1,900. All component-specific MS/MS parameters are given in Table S1. The UPLC-MS full scan mode was executed at capillary 1.5 kV and a cone voltage of 30V. An ACQUITY UPLC HSS T3 column (2.1 × 100 mm, 1.8 µm particle size) was used at a flow rate of 0.5 mL/min at 40°C. A gradient of acidified MilliQ-water (0.1% formic acid; solvent A) and acidified ACN (0.1% formic acid; solvent B) was used as a mobile phase starting at 10% B and rising to 100% B in 20 min. Solvent B was kept at 100% for 4 min before going to the initial ratio in 1 min and kept at 10% for 2 more min. Fig. S1 presents the structures of syringotoxin and fuscopeptin, along with the predicted structure of asplenin.

### Chemical CLiP hydrolysis

A 0.1 M NaOH stock solution (pH ~ 12) was freshly prepared. To generate a hydrolysis buffer within the target pH range, this NaOH solution was added dropwise to deionized water until the pH stabilized at approximately 11. This solution was then used as the controlled hydrolysis buffer. CLiP extracts were dissolved in the prepared buffer. Following solubilization, the pH was checked and, if necessary, re-adjusted to pH 11 by further dropwise addition of 0.1 M NaOH under constant stirring. To monitor the progress of hydrolysis, aliquots were taken hourly and analyzed by UPLC-MS/MS. Complete hydrolysis was confirmed by UPLC-MS/MS analysis.

### Assessing the impact of *R. solani* fungal biomass and metabolites on CLiP production

To test the effect of *R. solani* fungal biomass or SN on the CLiP production or inhibition, three different tests were performed.

#### Effect of *R. solani* sterile SN on CLiP production in PDB

Ten plugs of *R. solani* were incubated in baffled flasks containing PDB for 5 days at 18°C with shaking at 150 rpm and a 15% filling volume. The resulting SN was filter sterilized and mixed with fresh PDB in a 1:1 ratio. A blank treatment (blank1) consisted of sterile water that was combined with PDB in a 1:1 ratio. Bacterial strains were inoculated into the respective PDB mixtures and incubated for 48 h at 18°C with shaking at 150 rpm. After incubation, samples were centrifuged at 1,000 rpm for 10 min, filter sterilized, and analyzed for CLiP production using UPLC-MS/MS.

#### Effect of *R. solani* plugs on CLiP production in PDB

Ten plugs of *R. solani* were simultaneously added to PDB along with the bacterial strains. The mixtures were incubated for 48 h at 18°C with shaking at 150 rpm. The blank (blank2) treatment consisted of PDB. Following incubation, the samples were centrifuged at 1,000 rpm for 10 min, filter sterilized, and subsequently analyzed for CLiP production using UPLC-MS/MS.

#### Effect of *R. solani* SN on bacterial SN

To assess the effect of fungal metabolites on CLiP concentrations without impacting bacterial production levels, PDB containing 10 mycelium plugs of *R. solani* AG 1-IA or AG 2-2 was incubated at 18°C for 5 days. Following incubation, samples were filter sterilized.

In a parallel preparation, PDB was inoculated with *P. fuscovaginae* UPB0736 and incubated for 2 days at 18°C. The resulting bacterial SN was filter sterilized and subsequently mixed 1:1 with the filter-sterilized fungal SN, after which the mixture was analyzed. To assess whether the metabolites of *R. solani* AG 1-IA are of enzymatic origin, *R. solani* SN was also exposed to heat for 10 min at 100°C. This heat treatment is expected to denature proteins and thereby eliminate enzymatic activity. To test the influence of a higher incubation temperature of *R. solani*, the fungus was incubated for 2 or 5 days at 18°C or 28°C prior to further treatment.

### Antifungal test against *R. solani* AG 1-IA and AG 2-2 on PDA

The mix containing filter-sterilized *R. solani* SN and filter-sterilized bacterial SN was tested *in vitro* to evaluate if there is a loss in antifungal activity against mycelium growth of *R. solani* due to CLiP degradation. For this, the mixtures were mixed in a 1:1 ratio with double strength PDA and poured in sterile six-well petri plates. When the agar was cooled down, a mycelium plug of *R. solani* AG 1-IA STMX04-3 or *R. solani* AG 2-2 CuHav-Rs18 was placed in the center of the wells and incubated at 28°C for 24 or 48 h. The mycelium area was measured using ImageJ software and was used to determine the growth inhibition relative to the control.

### Phytotoxicity test on chicory leaves

The test strains were cultivated overnight in KB. Both whole broth cultures and cell-free filtrates were prepared. To obtain cell-free filtrates, cultures were centrifuged, and the SN was filter sterilized using a 0.22 µm membrane. For broth-based treatments, the KB broth was diluted with sterile water to achieve an optical density (OD) of 0.1, measured using an Infinite 200 Pro M Plex spectrophotometer (Tecan, Switzerland). For the treatment conditions, sterile cell-free culture medium (blank control) or SN from *R. solani* AG 1-IA or AG 2-2 (cultivated for 5 days in PDB at 18°C with agitation at 150 rpm and subsequently filter sterilized) were mixed with the bacterial samples or cell-free filtrates in a 1:1 ratio. A 300 µL aliquot of each treatment was injected into the adaxial surface of chicory leaves using a 1 mL syringe. The inoculated leaves were then placed individually on inverted 65 mm petri dishes within plastic trays containing a moistened cotton sheet saturated with 80 mL of sterile water to maintain humidity. Immediately after inoculation, the plastic trays were sealed with plastic wrap to retain humidity. The samples were then incubated at 28°C for 24 h prior to assessment. For each experiment, three leaves were used per treatment.

### CLiP degradation potential of *R. solani* isolates from a range of different anastomosis groups

*R. solani* isolates representing various AGs were initially cultured on PDA for 5 days at 28°C. Subsequently, 10 mycelial plugs from each isolate were transferred to 250 mL baffled flasks containing PDB, incubated at 28°C for 5 days under shaking conditions (150 rpm) with a 15% filling volume. After incubation, the culture SNs were filter sterilized and mixed in a 1:1 ratio with filter-sterilized SN from a separate PDB culture of *P. fuscovaginae* UPB0736, grown for 2 days at 18°C. The resulting mixtures were subjected to solid-phase extraction using Oasis HLB cartridges, and the extracts were analyzed by UPLC-MS/MS to assess CLiP stability and degradation.

### Statistical analysis

Statistical analyses were performed using R software version 2023.09.1 (37). For comparison between treatment groups, normality and homoscedasticity were checked with Shapiro-Wilk’s and Levene’s test (*P* = 0.05). The evaluation results of normally distributed data were analyzed using Tukey’s range test (homoskedastic) or Welch’s analysis of variance (heteroskedastic; *P*=0.05). Data that were not normally distributed were analyzed with the Kruskal-Wallis rank sum test and the pairwise Wilcoxon rank sum test. In other cases, the unequal variance *t*-test was used. In all cases, significances were only accepted if *P* < 0.05.

## RESULTS

### *R. solani* AG 1-IA STMX04-3 and AG 2-2 CuHav-Rs18 suppress syringotoxin production by *P. fuscovaginae* UPB0736

**Fig 1:**
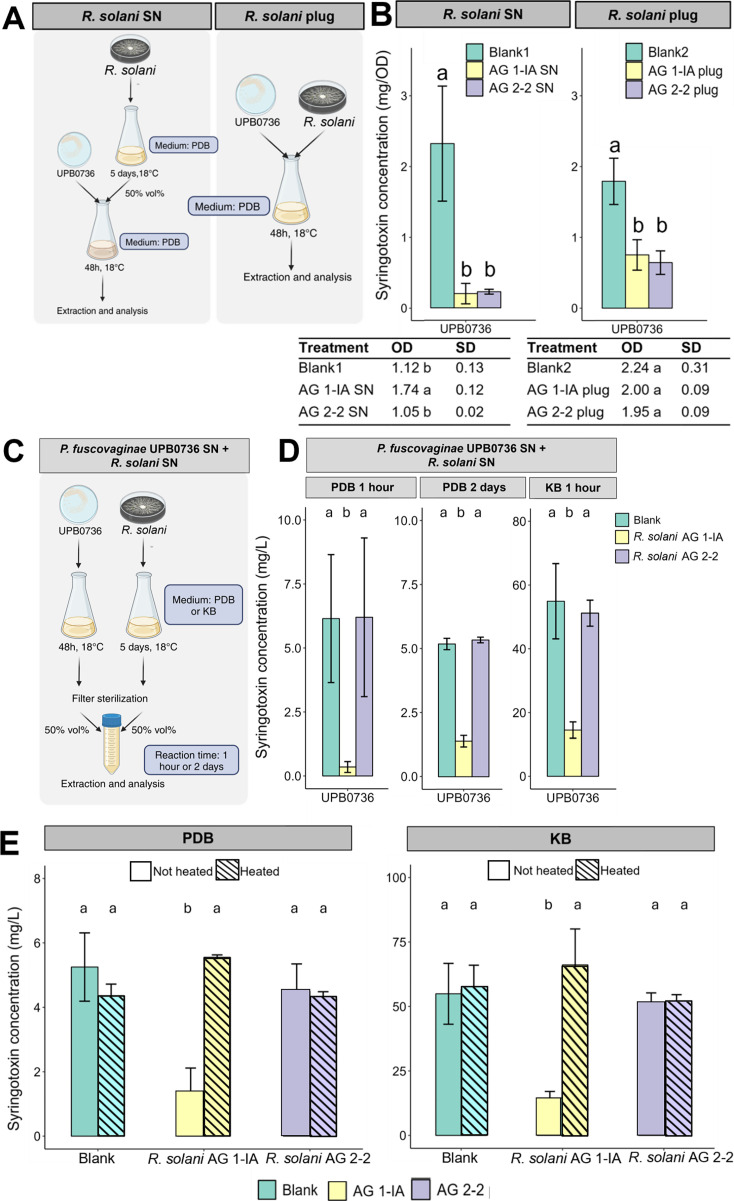
(**A**) Methodology for testing the effect of *R. solani* SN or plugs in the growing medium on syringotoxin production *by P. fuscovaginae* UPB0736. (**B**) Influence of *R. solani* SN and plugs on syringotoxin production (corrected for bacterial growth [OD_620_]) in mg/OD during incubation. Blank 1 contains 50% PDB and 50% water, and blank 2 contains 100% PDB. Significances were calculated using the pairwise Wilcoxon rank sum test. Bars with the same letters are not significantly different from each other (*P* < 0.05, *n* = 3). The OD values are given in the tables. Significances were calculated using the pairwise *t*-test (*P* < 0.05, *n* = 3). (**C**) Methodology for testing the effect of *R. solani* SN on syringotoxin in the SN of *P. fuscovaginae* UPB0736. (**D**) Influence of *R. solani* SN on syringotoxin concentration. Two separate experiments were conducted: in the first experiment, the concentrations were analyzed after 1 h, and in the second experiment, the concentrations were analyzed after 2 days (in static conditions). Significances were calculated using the pairwise Wilcoxon rank sum test. Bars with the same letters are not significantly different from each other (*P* < 0.05, *n* = 3). (**E**) The experiment was repeated in PDB and KB, but in addition, the *R. solani* SN and PDB were subjected to heat treatment at 100°C for 10 min prior to mixing. Concentrations were measured 1 h post-mixing. The statistical significance of the effects on syringotoxin production was determined using the unequal variance *t*-test. Bars with the same letters are not significantly different from each other (*P* < 0.05, *n* = 3).

Co-incubation of *P. fuscovaginae* UPB0736 with either filter-sterilized fungal SN or mycelium plugs of *R. solani* AG 1-IA or AG 2-2 added to the medium (Fig. 1A) led to a substantial reduction in syringotoxin levels (corrected for bacterial growth [OD_620_]), with the most pronounced decrease observed in the SN treatments (Fig. 1B). Syringotoxin concentrations in both AG 1-IA SN and AG 2-2 SN treatments were significantly lower than in the blank treatment (blank1). While the growth of *P. fuscovaginae* was enhanced in the presence of *R. solani* AG1-IA SN, this was not observed with *R. solani* AG 2-2 SN (Fig. 1B). Accordingly, the decline in total syringotoxin likely reflects both growth inhibition and direct or indirect interference with syringotoxin synthesis. Global syringotoxin concentrations (mg/L) are given in Table S2. In contrast, *R. solani* AG 1-IA SN significantly reduced syringotoxin concentrations without affecting bacterial cell density, indicating that the suppression likely occurs via direct degradation or inhibition of CLiP production. Importantly, the addition of fungal plugs also led to a decrease in syringotoxin levels, without reducing bacterial growth. Fuscopeptin concentrations were not monitored due to the focus on the primary antifungal agent, syringotoxin.

### *R. solani* AG 1-IA degrades syringotoxin

To distinguish between inhibited production and active degradation of syringotoxin, filter-sterilized SN from *R. solani* AG 1-IA or AG 2-2 was mixed with culture SN from *P. fuscovaginae* UPB0736 pre-grown for 2 days (Fig. 1C). Two distinct experiments were conducted: in the first experiment, the syringotoxin concentrations were analyzed after 1 h, and in a second experiment, they were analyzed after 2 days (Fig. 1D). As early as 1 h post-incubation, a significant reduction in syringotoxin was observed exclusively in the presence of AG 1-IA metabolites, suggesting active degradation rather than inhibited synthesis. This effect was consistent across both PDB and KB media. Notably, heat inactivation of the AG 1-IA SN prior to mixing abolished syringotoxin degradation (Fig. 1E), advocating for the putative involvement of a heat-labile enzyme. In contrast, no degradation was detected in treatments with *R. solani* AG 2-2 SN, indicating that this isolate does not secrete syringotoxin-degrading enzymes in the tested conditions.

### *R. solani* AG 1-IA SN degrades syringotoxin and fuscopeptin

**Fig 2:**
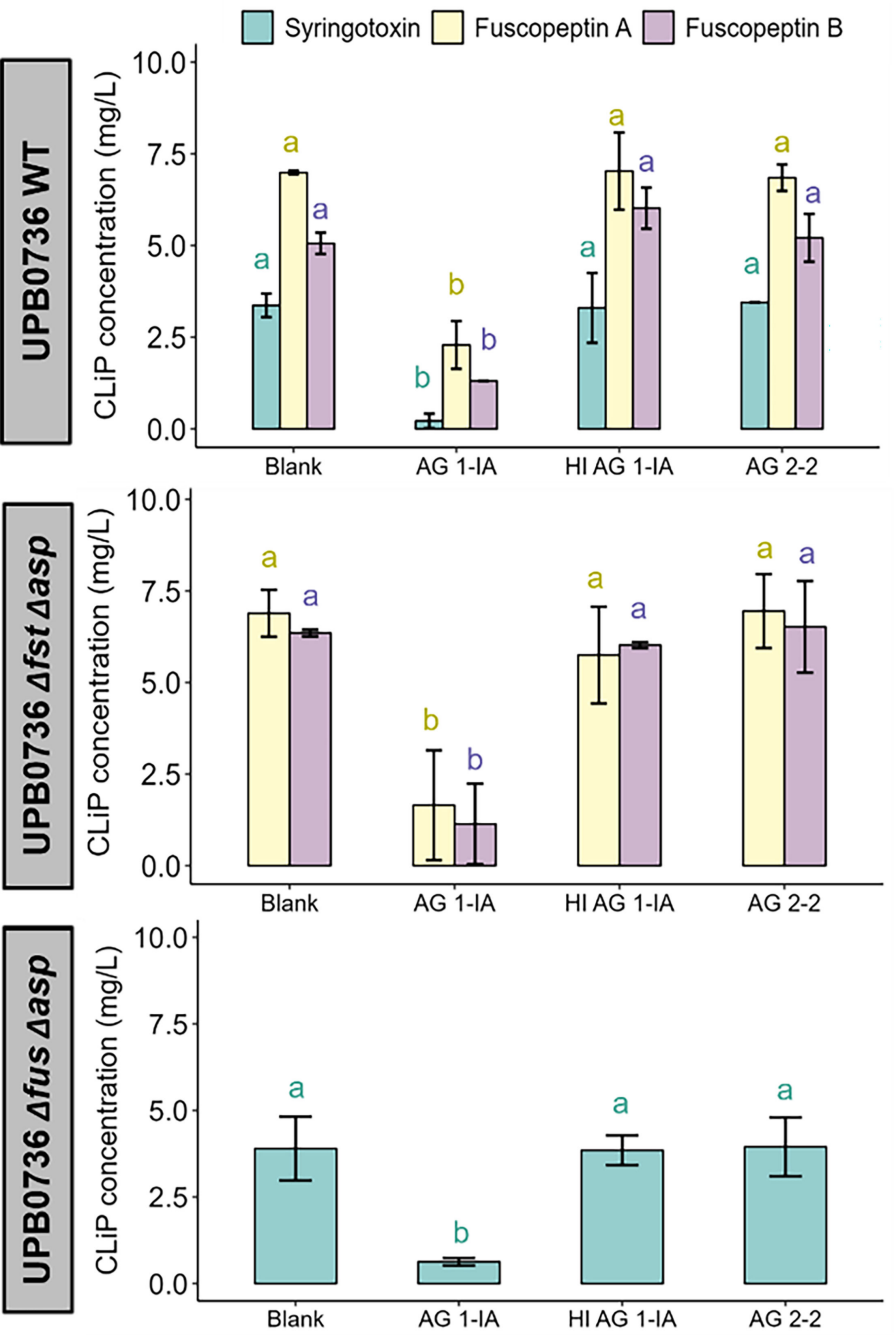
Influence of *R. solani* SN on CLiP concentrations of *P. fuscovaginae* UPB0736 and its double mutants in PDB. Significances were calculated using the pairwise Wilcoxon rank sum test. Bars with the same letters are not significantly different from each other (*P* < 0.05, *n* = 3).

The antifungal activity of the SN of *P. fuscovaginae* UPB0736 WT, the mutant that only produces syringotoxin (UPB0736*∆fus∆asp*), and the mutant that only produces fuscopeptin (UPB0736*∆fs*t*∆asp*) against *R. solani* was diminished upon exposure to AG 1-IA SN (Fig. S2; Table 2), correlating with a substantial reduction in syringotoxin and fuscopeptin levels as detected by multiple reaction monitoring (Fig. 2). Asplenin concentrations were not monitored due to the inability to perform accurate quantification. A full-scan MS analysis of the culture broths following treatment with *R. solani* AG 1-IA SN showed an accumulation of possible degradation products of syringotoxin and fuscopeptin A and B, each eluting at characteristic retention times. This pattern is likely consistent with the degradation of these CLiPs, supporting the involvement of AG 1-IA-secreted enzymes (Fig. S3).

**TABLE 2:** The relative fungal growth (%) and standard deviation (SD) of *R. solani* AG 1-IA and AG 2-2 in response to the (1:1) mixture of AG 1-IA SN, heat-inactivated (HI) AG 1-IA or AG 2-2 SN and *P. fuscovaginae* UPB0736, UPB0736*∆fst∆asp*, or UPB0736*∆fus∆asp* SN in a 1:1 ratio with double strength PDA

Treatment	Fungal growth^*c*^			
	AG 1-IA (%)	SD (%)	AG 2-2 (%)	SD (%)
Blank	100		100	
UPB0736 SN	13.6	4.0	9.5	1.2
UPB0736 SN + AG 1-IA SN	84.7*	6.9	75.1*	8.7
UPB0736 SN + HI AG 1-IA SN	12.9	1.5	10.2	1.5
UPB0736 SN + AG 2-2 SN	8.4	2.1	11.7	1.3
Blank	100		100	
UPB0736*∆fst∆asp*^*a*^ SN	15.2	1.4	19.3	5.0
UPB0736*∆fst∆asp* SN + AG 1-IA SN	89.6*	5.8	84.9*	10.2
UPB0736*∆fst∆asp* SN + HI AG 1-IA SN	17.3	2.1	20.1	3.3
UPB0736*∆fst∆asp* SN + AG 2-2 SN	9.6	4.3	16.8	4.4
Blank	100		100	
UPB0736*∆fus∆asp*^*b*^ SN	19.4	0.5	12.3	1.1
UPB0736*∆fus∆asp* SN + AG 1-IA SN	62.1*	2.4	91.7*	1.2
UPB0736*∆fus∆asp* SN + HI AG 1-IA SN	13.6	1.1	12.6	1.5
UPB0736*∆fus∆asp* SN + AG 2-2 SN	11.7	0.8	12.5	1.6

^*a*^ Syringotoxin- and asplenin-deficient mutant.

^*b*^ Fuscopeptin- and asplenin-deficient mutant.

^*c*^ Significant differences relative to the untreated UPB0736 SN were identified using Tukey’s range test and are indicated by an asterisk (*) (*P *< 0.05, *n *= 3).

### MS/MS determination of the hydrolysis product of syringotoxin

**Fig 3:**
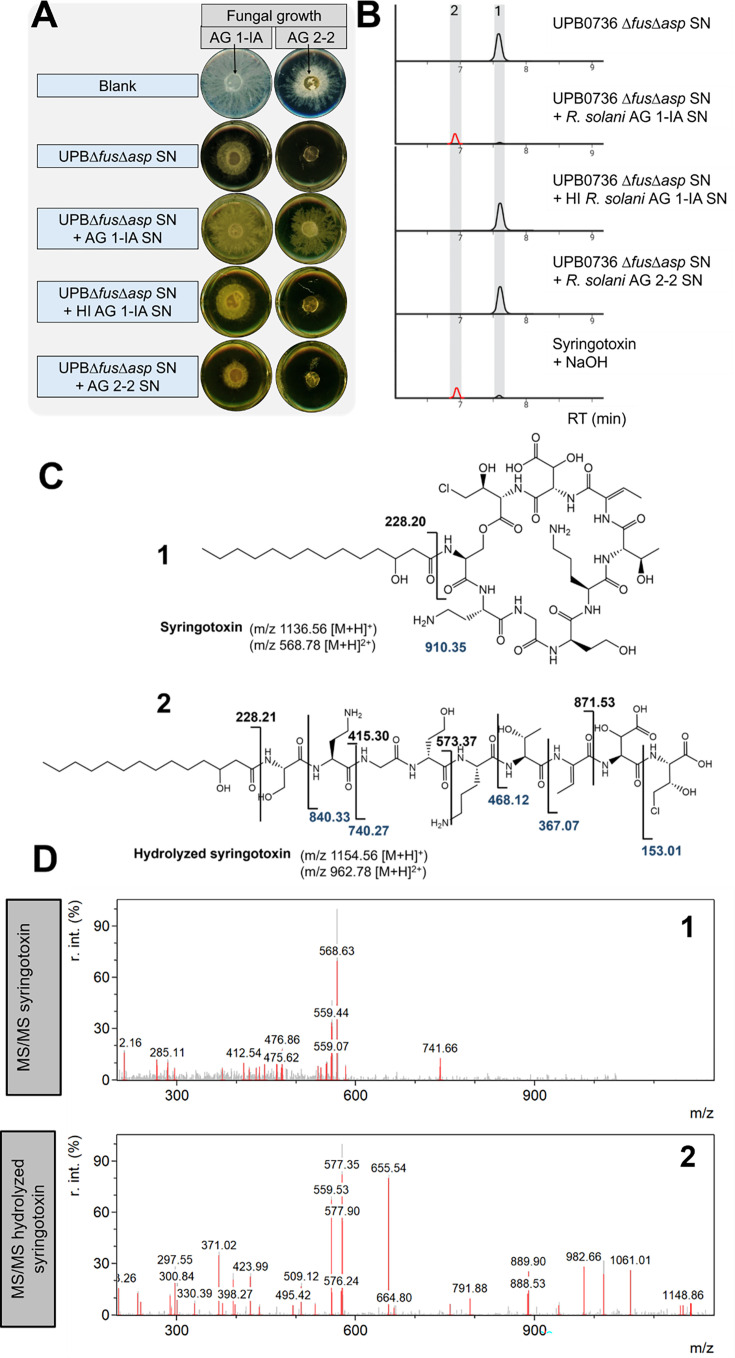
(**A**) The antifungal effect of the SN of the fuscopeptin- and asplenin-deficient mutant *P. fuscovaginae* UPB*∆fus∆asp* mixed with the unheated or heat inactivated (HI) SN of *R. solani* AG 1-IA or AG 2-2 on the mycelium growth of a plug of *R. solani* AG 1-IA STMX04-3 or AG 2-2 CuHav-Rs18. Blank treatments contained PDB instead of SN. (**B**) EIC for (1) syringotoxin (mass-to-charge ratio [m/z] 568.78, [M+2H]²^+^) and (2) hydrolyzed syringotoxin (m/z 577.78, [M+2H]²^+^). (**C**) Structures of (1) syringotoxin and (2) hydrolyzed syringotoxin. The m/z values ([M+H]^+^) of the matched MS/MS fragments are given. (**D**) MS/MS spectrum of the ions corresponding to (1) syringotoxin and (2) hydrolyzed syringotoxin. The fragmentation was done by collision-induced dissociation with an energy of 25V and a cone voltage of 30V.

To investigate the fate of syringotoxin in the presence of the *R. solani* SN, the mixtures containing filter-sterilized SN from *R. solani* AG 1-IA or AG 2-2 and the SN of *P. fuscovaginae* UPB*Δfus∆asp* (a mutant that only produces syringotoxin) were analyzed. As shown in Fig. 3A, mixtures containing AG 1-IA SN showed significantly reduced antifungal effect, indicating a structural modification of syringotoxin. In contrast, in heated samples (heat inactivated [HI]), the antifungal effect is diminished, showing that it might possibly be caused by enzymatic activity. Quantitative results of the antifungal effects are provided in Table 2.

A double-charged ion (m/z 577.78 [M + 2H]^2+^) is observed in the mixture containing *R. solani* AG 1-IA SN, which represents an 18 Da mass increase with respect to native syringotoxin (corresponding to +9 Da in the double-charged state). A more detailed MS analysis can be found in Fig. S4. This mass shift is consistent with hydrolysis of the ester bond within the cyclic structure of syringotoxin; however, cleavage of a peptide bond between two amino acids within the ring cannot be entirely ruled out as an alternative explanation. The formation of this hydrolysis product suggests the presence of a linearized form of syringotoxin, generated by ring-opening as a result of degradation activity from *R. solani* AG 1-IA. To support the identification of the hydrolyzed product, its retention time and MS spectrum were matched to those obtained by NaOH-induced hydrolysis of purified syringotoxin. Under these chemical conditions, hydrolysis is known to selectively target the ester bond that closes the macrocycle (28–40). The proposed chemical structures of syringotoxin and its hydrolyzed derivative are shown in Fig. 3C, and the corresponding MS/MS fragmentation patterns are presented in Fig. 3D. Nevertheless, the experimental MS/MS spectra of hydrolyzed syringotoxin show only weak agreement with the proposed fragments, which limits the reliability of sequence confirmation. While the similarities with chemical hydrolysis strongly suggest ester bond opening, enzymatic activity could, in principle, also generate cleavage at a peptide bond, leading to alternative linearized products. This ambiguity, combined with the limited fragment matching, should be considered when interpreting the structural assignment of the hydrolyzed product.

### *R. solani* AG 1-IA degrades fuscopeptin

**Fig 4:**
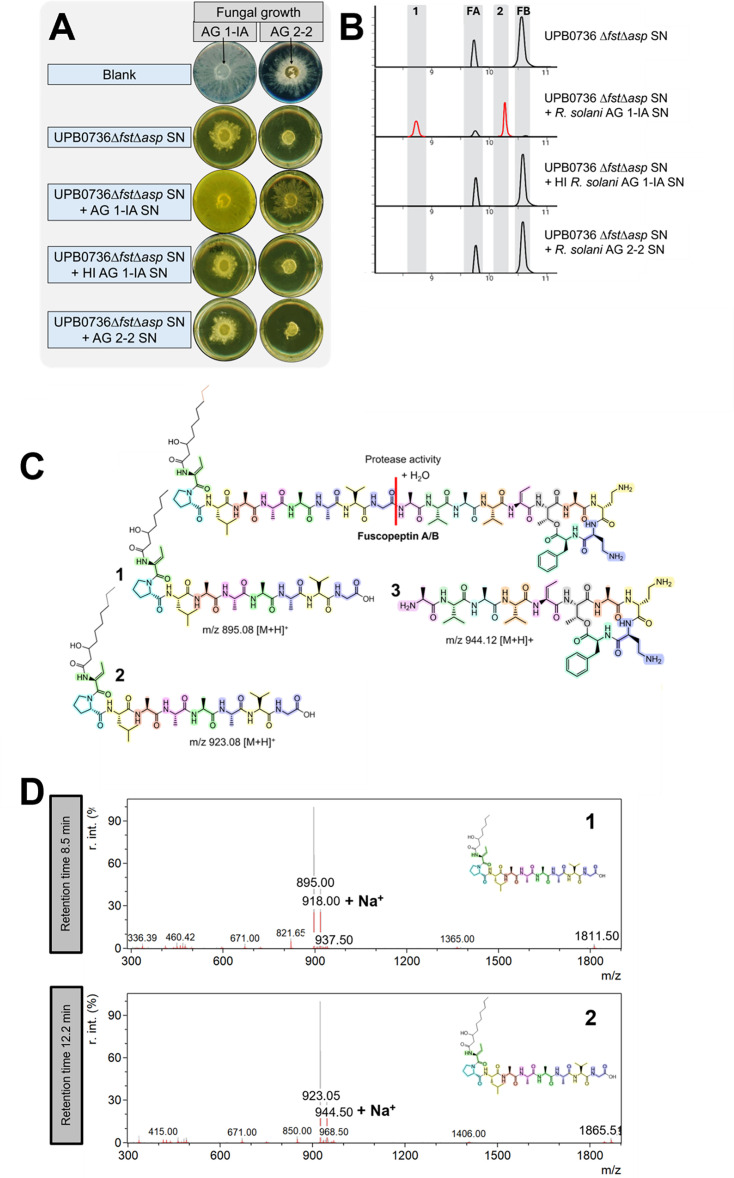
(**A**) The antifungal effect of the SN of the syringotoxin- and asplenin-deficient mutant *P. fuscovaginae* UPB*∆fst∆asp* mixed with the unheated or heat inactivated (HI) SN of *R. solani* AG 1-IA or AG 2-2 on the mycelium growth of a plug of *R. solani* AG 1-IA STMX04-3 or AG 2-2 CuHav-Rs18. Blank treatments contained PDB instead of SN. Please note that the blanks shown here are identical to those in Fig. 3A, as both originated from the same experimental setup. (**B**) EIC for fuscopeptin A (FA; mass-to-charge ratio [m/z] 910.02 [M + 2H]²^+^) (1) degradation product of FA (m/z 895.00 [M+H]^+^), fuscopeptin B (FB; m/z 924.04, [M + 2H]²^+^), and (2) degradation product of FB (m/z 924.05 [M + H]^+^), obtained from the analysis of culture extract mixtures. (**C**) Structures of fragments formed by proteolytic cleavage between glycine and alanine. (1) Degradation product of FA containing the fatty acid, (2) degradation product of FB containing the fatty acid, and (3) degradation product of FA and FB containing the lactone ring. (**D**) Observed MS fragments at retention times (1) 8.5 min and (2) 10.2 min, matching the degradation products of FA and FB, respectively.

**Fig 5:**
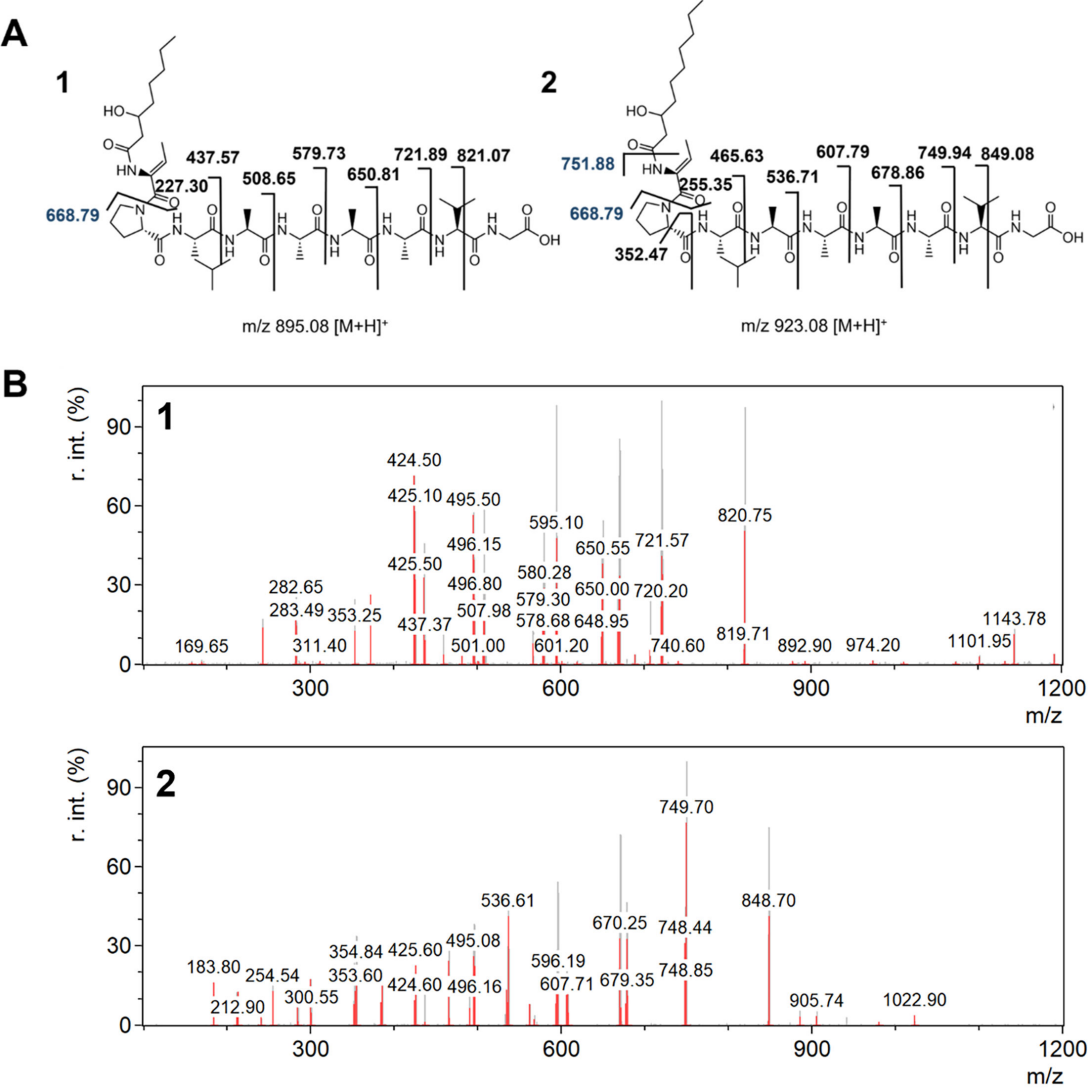
(**A**) Structure of the degradation product of fuscopeptin A (1) and the degradation product of fuscopeptin B (2). The mass-to-charge ratio (m/z) ([M + H]^+^) of the matched MS/MS fragments is given. (**B**) Observed MS/MS fragmentation patterns of (1) the degradation product of fuscopeptin A and (2) the degradation product of fuscopeptin B. The fragmentation was done by collision-induced dissociation with an energy of 25V and a cone voltage of 30V.

To investigate the fate of fuscopeptin A and B in the presence of fungal SN, the mixtures containing filter-sterilized SN from *R. solani* AG 1-IA or AG 2-2 and the SN of *P. fuscovaginae* UPB*ΔfstΔasp*, a mutant that only produces fuscopeptin, were analyzed. As shown in Fig. 4A, mixtures containing AG 1-IA SN showed significantly reduced antifungal activity, indicating structural modification of fuscopeptin. In contrast, in heated samples (HI), the antifungal effect is diminished. Quantitative results are provided in Table 2. The presence of AG 1-IA SN leads to the formation of chromatographic peaks at retention time 8.5 and 10.2 min, respectively (Fig. 4B). Further analysis of the MS spectra suggests that the observed peaks could possibly be attributed to enzymatically degraded forms of fuscopeptin A and fuscopeptin B. Specifically, a protease appears to cleave fuscopeptin at the peptide bond between the glycine and alanine residues, generating degradation products with m/z 895 [M + H]^+^ and m/z 923 [M + H]^+^ for fuscopeptin A and B, respectively. This enzyme seems to be inactivated by a heat treatment (HI). The enzymatic cleavage site and the observed MS fragments of the peaks at retention times 8.5 min and 10.2 min are given in Fig. 4C and D. Furthermore, the MS/MS fragments were compared with the theoretical fragmentation patterns derived from the chemical structures (Fig. 5A and B). This confirms that the peaks at 8.5 and 10.2 min in the chromatogram likely correspond to the degradation products of fuscopeptin A and fuscopeptin B, respectively. A peak with a mass corresponding to the complementary fragment resulting from the cleavage of fuscopeptin A and B (Fig. 4C) (3) was detected at a retention time of 3.4 min. However, MS/MS fragmentation data for this ion could not be obtained. No evidence was found for additional hydrolysis of the ester bond in fuscopeptin, which would result in the theoretical detection of a component with m/z 962.13 [M + H]^+^. The MS and MS/MS spectra of fuscopeptin A and B, chemically hydrolyzed using NaOH, in which lactone ring opening results in a linearized structure, are shown in Fig. S5. The fragmentation patterns of these hydrolyzed standards did not match the peaks observed in our samples, confirming that *R. solani* does not degrade fuscopeptin by lactone ring cleavage.

### Higher temperatures enhance degradation of CLiPs by *R. solani* AG 1-IA

**Fig 6:**
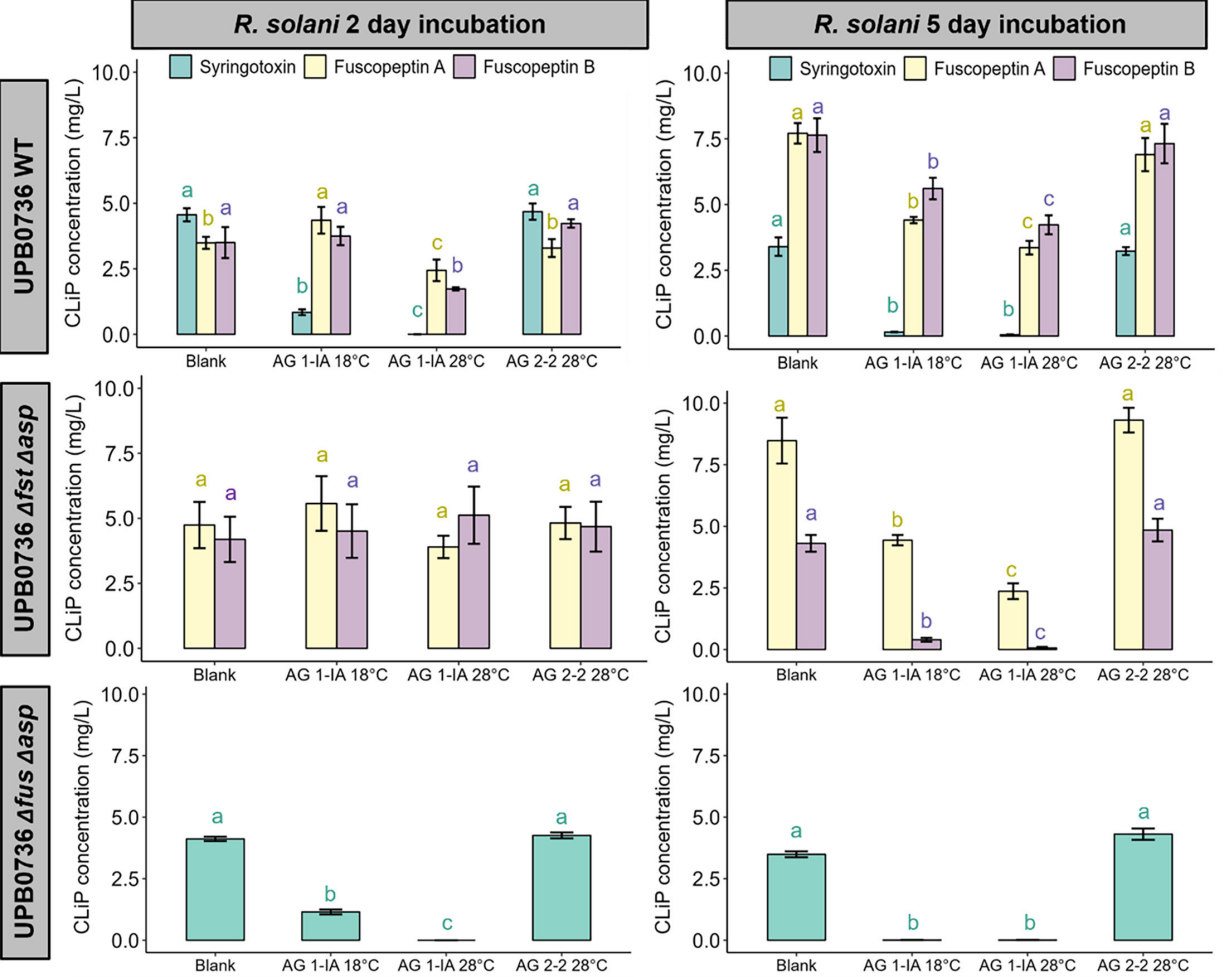
Influence of *R. solani* SN on CLiP concentration. Ten mycelium plugs of *R. solani* AG 1-IA STMX04-3 or AG 2-2 CuHav-Rs18 were incubated in PDB for 2 or 5 days at 18°C and 28°C. The filter-sterilized SN was mixed in a 1:1 ratio with filter-sterilized PDB that was previously inoculated with the bacterial strains of *P. fuscovaginae* UPB0736 at 18°C for 2 days. The concentrations were analyzed after 1 h reaction time with UPLC-MS/MS. Significance of effects on concentration was calculated separately for each CLiP using the pairwise Wilcoxon rank sum test. Bars with the same letters are not significantly different from each other (*P* < 0.05, *n* = 3).

*R. solani* AG 1-IA exhibits optimal growth at 28°C compared to 18°C, raising the hypothesis that CLiP degradation may proceed more rapidly at elevated temperatures. To investigate this, *R. solani* AG 1-IA was incubated at 18°C or 28°C for either 2 or 5 days in liquid PDB. These four conditions were subsequently assessed for the ability to degrade syringotoxin and fuscopeptin. A schematic overview of the experimental setup is provided in Fig. S6, and the corresponding CLiP quantification results are shown in Fig. 6.

Degradation of syringotoxin and fuscopeptin was more pronounced after incubation with *R. solani* AG 1-IA SN at 28°C compared to 18°C, indicating increased degradation activity at this temperature. Syringotoxin was generally more susceptible to degradation than fuscopeptin, and after 5 days, all AG 1-IA treatments resulted in CLiP degradation. In contrast, *R. solani* AG 2-2 did not degrade CLiPs at either 18°C or 28°C. This temperature-mediated effect is likely driven by the higher metabolic activity of *R. solani* AG 1-IA at 28°C, which may stimulate increased production, secretion, and/or catalytic efficiency of the extracellular enzymes responsible for CLiP hydrolysis, ultimately accelerating the breakdown of syringotoxin and fuscopeptin under warmer conditions.

### Asplenin is not affected by *R. solani*

To assess the impact of *R. solani* AG 1-IA and AG 2-2 SN on asplenin, the *P. fuscovaginae* mutant strain UPB∆*fst*∆*fus*, which only produces asplenin, was employed. Additionally, a control treatment involving chemical hydrolysis of asplenin using NaOH was included. Exposure to AG 1-IA and AG 2-2 SN had no observable effect on asplenin, indicating resistance to degradation under these conditions. In contrast, chemical hydrolysis of the ester bond resulted in distinct chromatographic peaks corresponding to hydrolyzed asplenin, with a mass shift of +18 Da (or +9 Da in the double-charged state), confirming successful ring opening (Fig. S7).

### The phytotoxicity of *P. fuscovaginae* UPB0736 SN on chicory is neutralized by *R. solani* AG 1-IA

**Fig 7:**
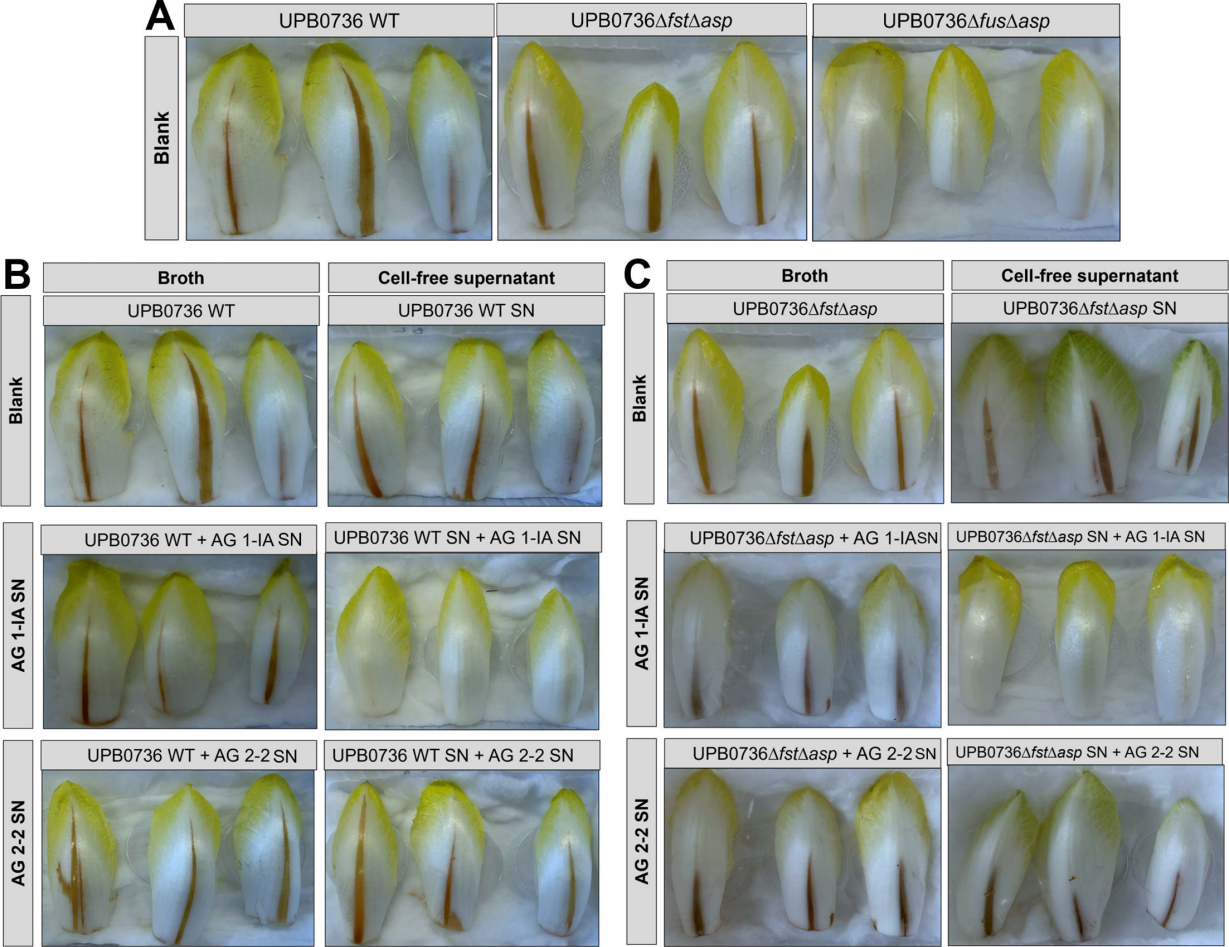
Phytotoxic effect of (**A**) *P. fuscovaginae* UPB0736 WT broth, broth of a mutant that only produces fuscopeptin (UPB0736*∆fst∆asp*), and broth of a mutant that only produces syringotoxin (UPB0736*∆fus∆asp*) injected in chicory leaves. (**B**) Treatments consisted of UPB0736 WT broth in KB or cell-free SN mixed in a 1:1 ratio with either sterile PDB as a blank control or filter-sterilized SN of *R. solani* AG 1-IA STMX04-3 or AG 2-2 CuHav-Rs18. (**C**) Treatments consisted of UPB0736*∆fst∆asp* KB broth or cell-free SN mixed in a 1:1 ratio with either sterile PDB as a blank control or filter-sterilized SN of *R. solani* AG 1-IA or AG 2-2. Please note that the broth blanks for panel B (UPB0736 WT) and panel C (UPB0736*∆fst∆asp*) were the same as the ones used in panel A, as both originated from the same experimental setup.

The phytotoxic effects of *P. fuscovaginae* UPB0736, the mutant that only produces syringotoxin (UPB0736∆*fus*∆*asp*), and the mutant that only produces fuscopeptin (UPB0736∆*fst*∆*asp*) were demonstrated on chicory leaves. Both the bacteria and the cell-free filter-sterilized SN were tested. The results indicate that the phytotoxic effect in chicory is mainly due to fuscopeptin, since syringotoxin did not induce a phytotoxic effect in chicory leaves (Fig. 7A). Additionally, when the SN of *R. solani* AG 1-IA was combined with UPB0736 wild-type broth or SN, or UPB0736∆*fst*∆*asp* broth of SN, no phytotoxic activity was observed (Fig. 7B and C). This confirms that degradation by AG 1-IA neutralizes fuscopeptin’s phytotoxicity.

### CLiP degradation is a common trait among AG 1-IA isolates

To assess how widespread the trait to degrade CLiPs is among *R. solani* isolates, an additional collection of isolates from different anastomosis groups that were previously isolated in Vietnam was tested (35). Interestingly, all five tested AG 1-IA isolates from different regions in Vietnam were able to degrade both syringotoxin and fuscopeptin, employing the same degradation mechanisms previously observed for isolate AG 1-IA STMX04-3. In contrast, other multinucleate AG isolates did not affect syringotoxin, although some exhibited moderate degradation of fuscopeptin, likely by protease activity targeting the peptide bond between glycine and alanine. This indicates that degradation of CLiPs is a common trait among AG 1-IA, the major sheath blight pathogen on rice. Intriguingly, the two binucleate AGs tested (AG A and AG Fc/V) were also capable of degrading both CLiPs (Table 3); however, the mechanism of fuscopeptin degradation employed by AG A differed from the one described in this study. One possibility is the involvement of a distinct class of peptidases with different substrate specificities, or even a two-step degradation process in which an initial modification renders fuscopeptin more accessible to subsequent proteolytic cleavage. Although further work is required to pinpoint the exact mechanism, these preliminary differences highlight that *R. solani* may employ multiple biochemical strategies to neutralize CLiPs, depending on the AG and enzymatic repertoire. The measured concentrations in the samples are presented in Table S3, full MS-scans are shown in Fig. S8 and S9, and the formed MS spectra by AG 1-IA STMX04-4 at different retention times are given in Fig. S10. Based on the pH values of the *R. solani* SNs, which ranged between 4.52 and 7.00, with the second-lowest pH of 4.69 (AG 7) not inducing degradation, it can be concluded that pH alone has limited influence on CLiP stability (Table S4).

**TABLE 3:** Degradation potential of the SN from different *R. solani* isolates on syringotoxin and fuscopeptin^*a*^

AG	Host plant	Isolate	Syringotoxin	Fuscopeptin
1-IA	Chinese cabbage	STMX04-3	++	++
	Chinese cabbage	STMX04-4	++	++
		HNGL01-3	++	++
	Pak choi	STMX01-1	++	++
	Turnip cabbage	HNDD01-3	++	++
	White cabbage	CTCR01-3	++	++
1-IB	White cabbage	LDDL05-3	0	0
1-ID	Chinese cabbage	DNBH05-4	0	+
1-IG	Chinese cabbage	DNBH05-1-2	0	+
2-2	Bean	CuHav-Rs18	0	0
	Turnip cabbage	HNDA01-1	0	0
4-HGI		STST01-1	0	+
7	Turnip cabbage	HNDA02-1	0	0
A	Mustard cabbage	DNBH04-1	++	+
Fc/V	Chinese cabbage	LDDL02-1	++	++

^*a*^ Full degradation is indicated as ++, no degradation as 0, and partial degradation as +. See Fig. S8 to S10 for more details.

## DISCUSSION

In this study, co-incubation of *P. fuscovaginae* UPB0736 with sterile SNs or mycelial plugs of *R. solani* AG 1-IA or AG 2-2 led to a marked reduction in syringotoxin levels. For AG 2-2 SN, the effect was twofold: it inhibited bacterial growth and suppressed syringotoxin biosynthesis. Consistently, exposure to AG 1-IA and AG 2-2 plugs also lowered syringotoxin concentrations. Subsequent UPLC-MS/MS analysis revealed that this decrease was attributable not only to impaired biosynthesis but also to degradation, specifically by AG 1-IA. The degradation activity was abolished upon heat inactivation, suggesting the involvement of heat-labile enzymatic components. Specifically, *R. solani* AG 1-IA, but not AG 2-2, probably produces multiple enzymes that mediate the hydrolysis of syringotoxin and fuscopeptin, leading to the formation of linearized or fragmented, biologically inactive congeners, consistent with hydrolyzation of the ester bond (as observed for syringotoxin) or protease cleavage between the glycine and alanine amino acids (as observed for fuscopeptin).

The biosynthesis of CLiPs in *Pseudomonas* is tightly regulated by environmental cues, including quorum sensing, two-component regulatory systems, and LuxR-type regulators. Fungal-associated molecules may act as signals that upregulate CLiP production, as seen in various *Pseudomonas* strains (41, 42). In *Pseudomonas nunensis* (previously known as *P. fluorescens*) In5, for instance, fungal-associated molecules positively regulate the biosynthesis of the antifungal CLiPs nunamycin and nunapeptin by activating the LuxR-type regulator NunF. CLiP production by *P. nunensis* In5 is also activated by the global regulator GacA that is positively influenced by plant-derived signal molecules rather than fungal-associated molecules (43, 44). Also, CLiPs produced by *Pseudomonas* spp. often work synergistically with fungal cell wall-degrading enzymes, suggesting that the presence of fungal elements in the inoculation broth could enhance CLiP activity or production (45). In this work, however, the presence of *R. solani* or its metabolites caused an inhibition of CLiP production, rather than an increase. It is possible that certain fungal CLiP-degrading enzymes or signaling molecules influencing CLiP production are only expressed or activated during direct interaction between the fungus and the bacterium, such as during co-inoculation. More generally, a reduction in CLiP levels without evidence of direct degradation, as observed in the presence of *R. solani* AG 2-2, suggests that some *R. solani* isolates can suppress bacterial CLiP biosynthesis by the bacteria. This could imply that *R. solani*-derived metabolites may inhibit synthesis rather than enzymatically degrade CLiPs. This requires further investigation.

Enzymatic degradation of CLiPs produced by *Pseudomonas* and *Bacillus* has mainly been associated with gram-positive bacteria. To our knowledge, degradation of CLiPs by fungi has not been shown before. Although the specific CLiP-hydrolyzing enzymes produced by *R. solani* remain unidentified, this research proposes the involvement of at least two enzymatic activities: a lactonase or esterase able to open the lactone ring (or possibly another peptide sequence) in syringotoxin, and a protease that cleaves the glycine-alanine amino acid bond in the peptide backbone of fuscopeptin. In addition, proteolytic degradation of syringotoxin cannot be excluded but would require a distinct protease targeting alternative peptide bonds, as glycine-alanine residues are not present in syringotoxin. In general, the proposed mechanism remains hypothetical, as the responsible enzymes are still unknown. Identifying and characterizing these activities will be an important focus for future research.

It has been shown that actinobacteria of the *Mycetocola* genus can protect mushrooms from the mushroom pathogen *Pseudomonas tolaasii* by inactivating tolaasin, an antifungal CLiP and a primary virulence factor responsible for brown blotch disease in mushrooms. *Mycetocola tolaasinivorans* and *Mycetocola lacteus* neutralize tolaasin by cleaving the lactone of the toxin, leading to the formation of inactive linear congeners and suppressing the pathogenic activity of *P. tolaasii*. In addition to inactivating tolaasin, these helper bacteria can also degrade pseudodesmin, a CLiP that promotes the swarming motility of *P. tolaasii* (46). In another study, the enzymatic degradation of orfamide A, a CLiP synthesized by *P. protegens*, was investigated. The degradation process is initiated by *Rhodococcus globerulus* D757, a soil actinobacterium that catalyzes the hydrolysis of the ester bond within the macrocyclic structure of orfamide A, leading to the molecule’s linearization and subsequent inactivation, likely facilitated by esterase activity. Following this, *Stenotrophomonas indicatrix* D763 continues the degradation of the linearized orfamide A through proteolytic enzymes (47). It was also demonstrated that *Streptomyces sp. Mg1* hydrolyzes surfactin, leading to the formation of a linear variant of the molecule (48). *Streptomyces venezuelae* is also capable of degrading various CLiPs, including structurally diverse iturin and fengycin type components produced by *Bacillus*, as well as a range of structurally diverse CLiPs from *Pseudomonas* (e.g., sessilin, tolaasin, orfamide, xantholysin, and putisolvin). This degradation diminishes the biocontrol efficacy of these compounds. These enzymes likely include esterases, endo-proteases, and exo-proteases. The esterases and endo-proteases are believed to initiate the linearization of the cyclic structure of the CLiPs, while exo-proteases further degrade the linearized peptides into smaller fragments, such as free fatty acids and amino acids (49).

*P. fuscovaginae* causes sheath brown rot in rice, particularly in high-altitude regions. These regions are typically defined by cooler environmental temperatures, ranging between 18°C and 23°C or lower (15, 17, 19–21). Our previous research has shown that low temperatures enhance CLiP biosynthesis. At 18°C, *P. fuscovaginae* UPB0736 produces more syringotoxin and fuscopeptin, resulting in enhanced antifungal activity and phytopathogenic potential (34). This temperature-dependent increase in CLiP biosynthesis may contribute to the ecological success of *P. fuscovaginae* and the higher incidence of rice sheath brown rot in cooler, high-altitude environments. In contrast, *R. solani* AG 1-IA causes rice sheath blight, mainly in warm, humid environments, with peak infection rates occurring between 25°C and 30°C (3–7). It is also primarily found on the sheath and leaves, where it is exposed to higher temperatures and greater temperature fluctuations compared to a soil environment (50). At elevated temperatures (28°C), *R. solani* AG 1-IA showed enhanced enzymatic degradation of syringotoxin and fuscopeptin. This degradation reduced the antifungal and phytotoxic efficacy of these CLiPs. In our analysis, we found that all tested *R. solani* AG 1-IA isolates demonstrated the ability to degrade both syringotoxin and fuscopeptin. This finding reinforces the hypothesis that both *R. solani* AG 1-IA and *P. fuscovaginae* UPB0736 might inhabit the same ecological niche, the rice sheath, and possibly engage in direct antagonism. Since their simultaneous occurrence under field conditions has, to the best of our knowledge, not been documented, it raises the question of whether temperature-dependent dynamics in CLiP production and enzymatic degradation determine which pathogen dominates. Rather than coexisting, these pathogens may outcompete one another depending on the environmental context: at lower temperatures, enhanced CLiP production by *P. fuscovaginae* may inhibit *R. solani* AG 1-IA, while at higher temperatures, CLiP degradation by *R. solani* AG 1-IA may suppress bacterial virulence. The consistent ability of AG 1-IA isolates to neutralize CLiPs, particularly pronounced at elevated temperatures (28°C), supports the idea that this antagonistic interaction is not incidental but may reflect an evolved ecological strategy to displace competing sheath pathogens under favorable conditions. Further research should now move from the lab to the field to explore these interactions under realistic growing conditions.

Other multinucleate AGs besides AG 2-2, including AG 1-IB, AG 1-ID, AG 1-IG, AG 4-HGI, and AG 7, do not exhibit the ability to degrade syringotoxin. Interestingly, some AGs, such as AG 1-ID, AG 1-IG, and AG 4-HGI, demonstrate a moderate capacity to degrade fuscopeptin, further supporting the notion that distinct enzymes mediate the degradation of syringotoxin and fuscopeptin. Notably, all tested multinucleate isolates appear to utilize the same degradation mechanisms as AG 1-IA. AG 1-IB, AG 2-2, and AG 7 were not able to degrade syringotoxin and fuscopeptin. Remarkably, the degradation of syringotoxin and fuscopeptin was also observed within the two tested binucleate AGs, AG A and AG Fc/V. A wider range of binucleate AGs should be tested to check if the capacity to degrade CLiPs is widespread among them.

Given the demonstrated ability of *R. solani* AG 1-IA, AG A, and AG Fc/V isolates to enzymatically degrade syringotoxin and fuscopeptin, it is of considerable scientific interest to investigate whether these fungal isolates can also degrade CLiPs from the Mycin and Peptin families produced by other *Pseudomonas* species. Such studies could reveal potential co-evolutionary adaptations among plant pathogens occurring in the same niche, reflecting antagonistic interactions and niche competition mediated by targeted CLiP degradation. Variants of the Mycin and Peptin families are produced by a wide range of *Pseudomonas* species, particularly within the *P. syringae* and *P. fluorescens* groups. Multiple phylogroups of *P. syringae* have been shown to synthesize mycin-type CLiPs such as syringomycin, syringotoxin, syringostatin, and pseudomycin, alongside peptin-type CLiPs including syringopeptins SP22 and SP25, cichopeptin, and cichorinotoxin. Similarly, members of the *P. fluorescens* group are known to produce diverse mycins (e.g., cormycin, syringomycin, thanamycin, nunamycin, and keanumycin) and peptins (e.g., jessinipeptin, corpeptin, nunapeptin, thanapeptin, and braspeptin) (33, 43). Notably, syringotoxin production has been observed in specific *P. syringae* pv. *syringae* and *P. syringae* pv. *lapsa* isolates. The peptin that is produced by these isolates is not fuscopeptin but SP25 or an SP25 variant (33). It remains to be investigated whether *R. solani* isolates capable of degrading fuscopeptin can also hydrolyze SP25 or other peptins. Future studies could reveal broader patterns of co-evolution between CLiP-producing bacteria and fungi occupying the same ecological niches and shed light on the molecular strategies by which microbial competitors neutralize each other’s bioactive metabolites. Such efforts may further elucidate the co-evolutionary dynamics underlying microbial antagonism and niche competition, potentially uncovering horizontal gene transfer events or selective pressures that have shaped the evolution of CLiP biosynthesis in *Pseudomonas* species and the emergence of CLiP-degrading or CLiP biosynthesis-inhibiting capabilities in specific *R. solani* lineages.

## SUPPLEMENTAL MATERIAL

Supplemental material aem.01524-25-s0001.docx Fig. S1 to S10; Tables S1 to S4.

ASM does not own the copyrights to Supplemental Material that may be linked to, or accessed through, an article. The authors have granted ASM a non-exclusive, world-wide license to publish the Supplemental Material files. Please contact the corresponding author directly for reuse.
