# Supplementary material for: Warfare among rice sheath pathogens: Rhizoctonia solani AG 1-IA neutralizes Pseudomonas fuscovaginae cyclic lipopeptides
Source: Appl Environ Microbiol. 2026 Jan 16;92(2):e01524-25. doi: 10.1128/aem.01524-25 (PMC12915298; doi:10.1128/aem.01524-25)
Supplement: Supplemental material — Fig. S1 to S10; Tables S1 to S4. [file aem.01524-25-s0001.docx]

**Supplemental material**

**Warfare among rice sheath pathogens: *Rhizoctonia solani* AG 1-IA neutralizes *Pseudomonas fuscovaginae* cyclic lipopeptides**

Jasmine De Rop^1,2^, Durga Prasad^3^, Niels Geudens^3^, Lu Zhou^2^, Pieter Spanoghe^1^, José C. Martins^3^, Monica Höfte^2*^

^1^ Lab of [Crop Protection Chemistry](https://www.ugent.be/bw/plants-and-crops/en/research/cropprotectionchemistry/overview.htm), Department of Plants and Crops, Faculty of Bioscience Engineering, Ghent University, Ghent, Belgium

^2^ Lab of [Phytopathology](https://www.ugent.be/bw/plants-and-crops/en/research/phytopathology/overview.htm), Department of Plants and Crops, Faculty of Bioscience Engineering, Ghent University, Ghent, Belgium

^3^ NMR and Structure Analysis Unit, Department of Organic and Macromolecular Chemistry, Faculty of Sciences, Ghent University, Ghent, Belgium

*****Corresponding author

e-mail address: monica.hofte@ugent.be


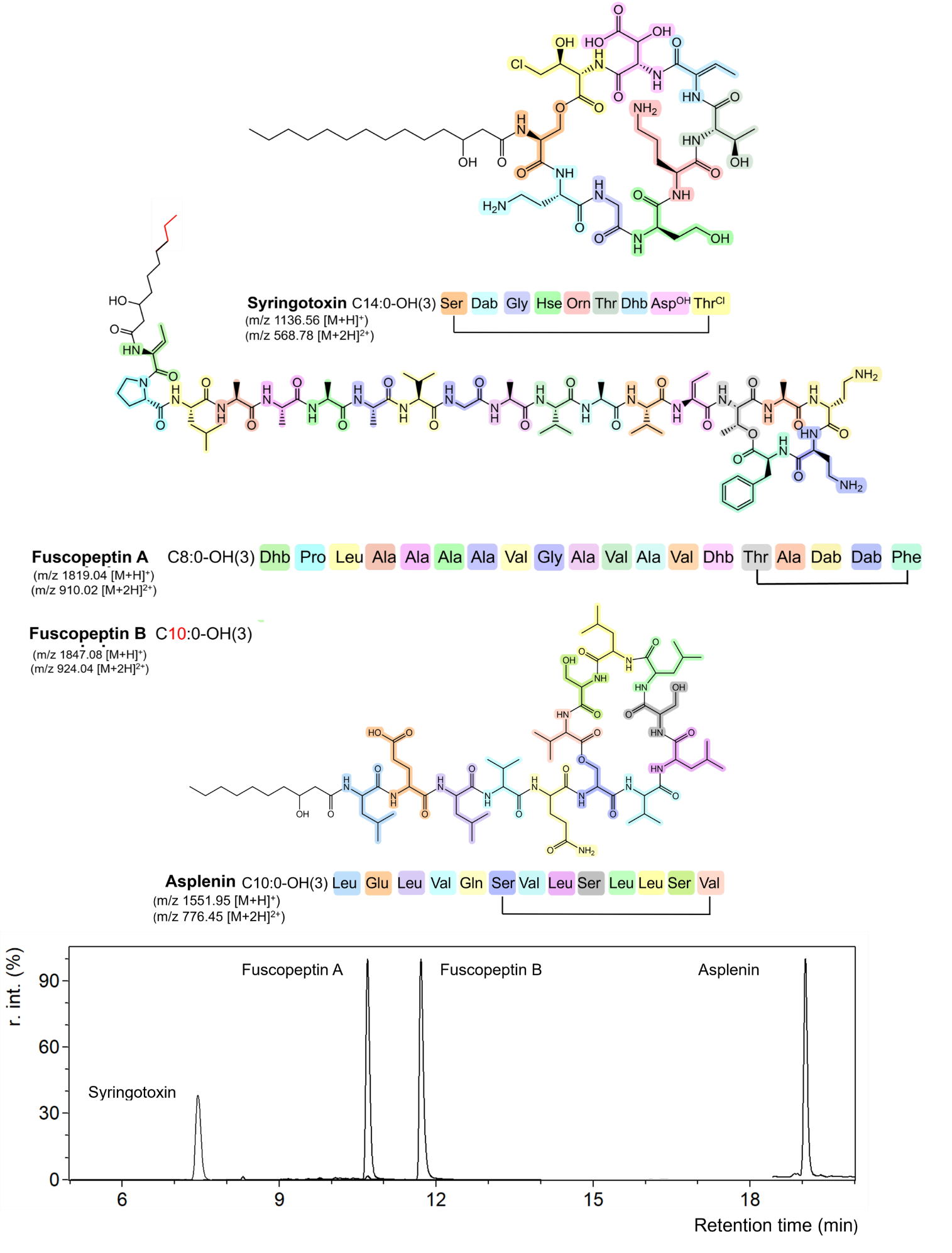


**Figure S1.** Chemical structures of syringotoxin, fuscopeptin A, fuscopeptin B and asplenin.


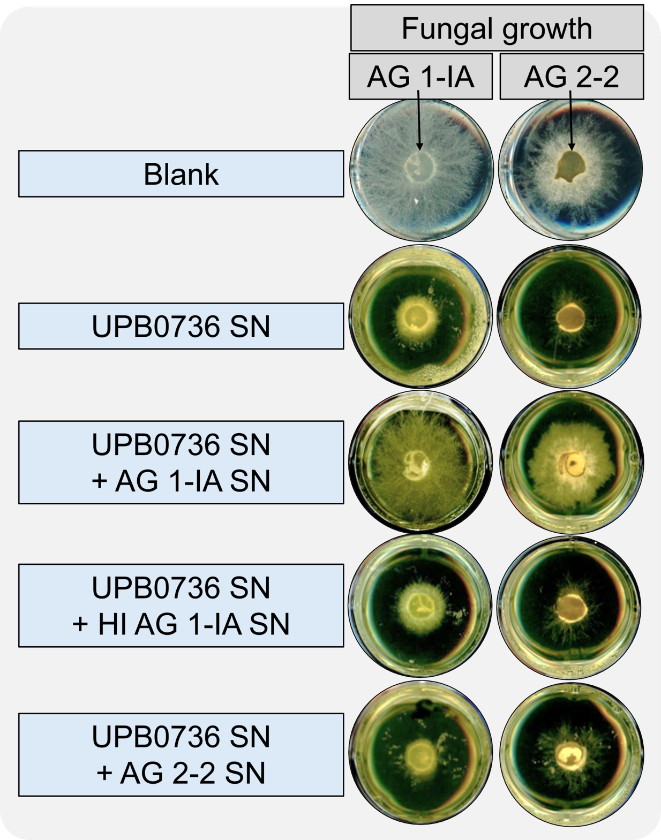


**Figure S2.** The antifungal effect of *P. fuscovaginae* UPB0736 WT supernatant (SN) mixed with the SN of *R. solani* AG 1-IA or AG 2-2, against the mycelium of *R. solani* AG 1-IA and AG 2-2. Heat inactivation (HI) of the *R. solani* AG 1-IA SN led to the restoration of the biocontrol activity.


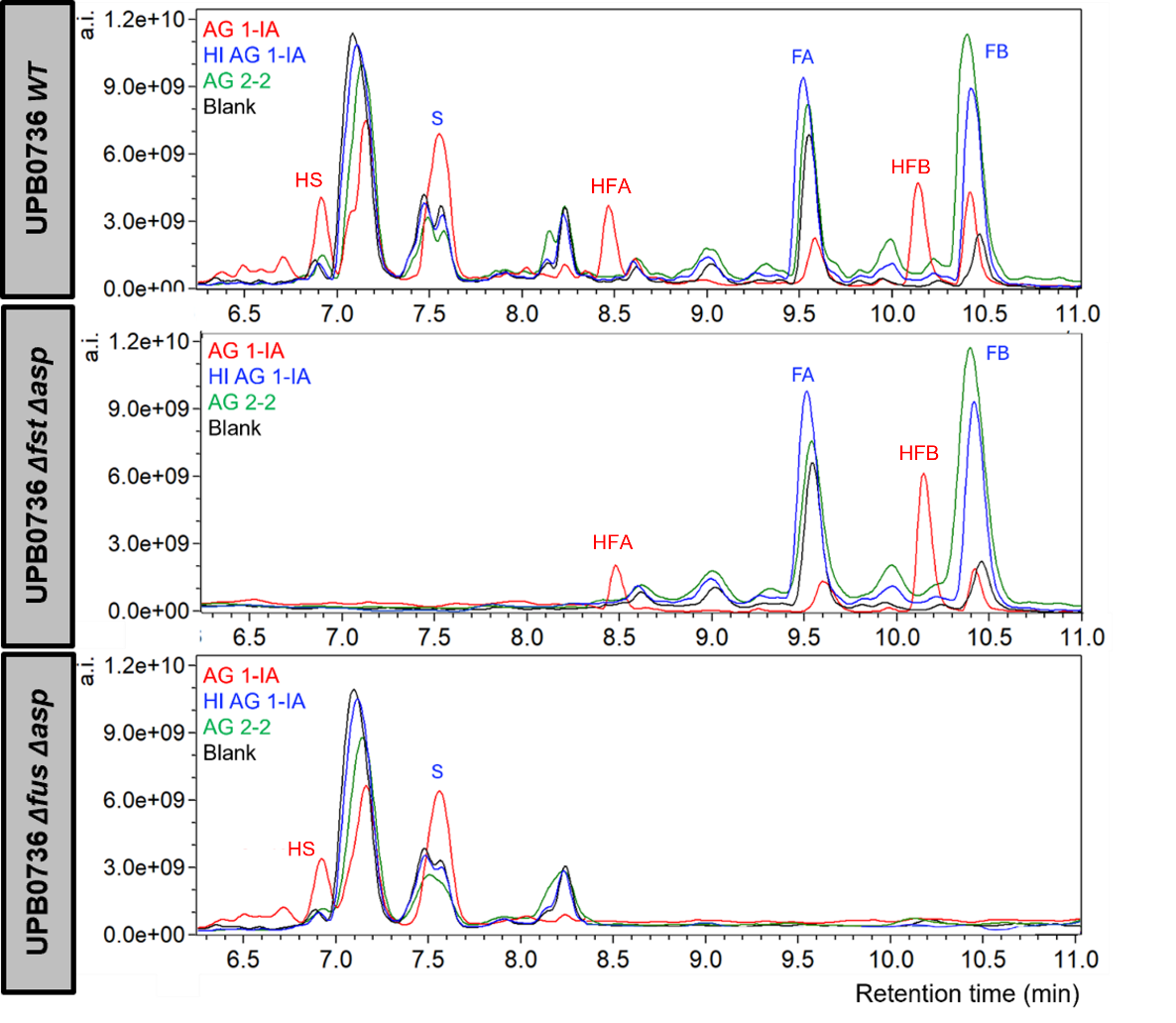


**Figure S3.** UPLC-MS scan (cone 30V) of the mixture of *Pseudomonas fuscovaginae* UPB0736 WT supernatant (SN), the fuscopeptin producing mutant UPB0736*∆fst∆asp* SN and the syringotoxin producing mutant UPB0736*∆fus∆asp* SN with R. solani AG 1-IA SN, heat inactivated (HI) AG 1-IA SN or AG 2-2 SN. Different CLiPs could be identified (S: syringotoxin, HS: hydrolyzed syringotoxin, FA: fuscopeptin A, HFA: hydrolyzed fuscopeptin A, FB: fuscopeptin B, HFB: hydrolyzed fuscopeptin B).


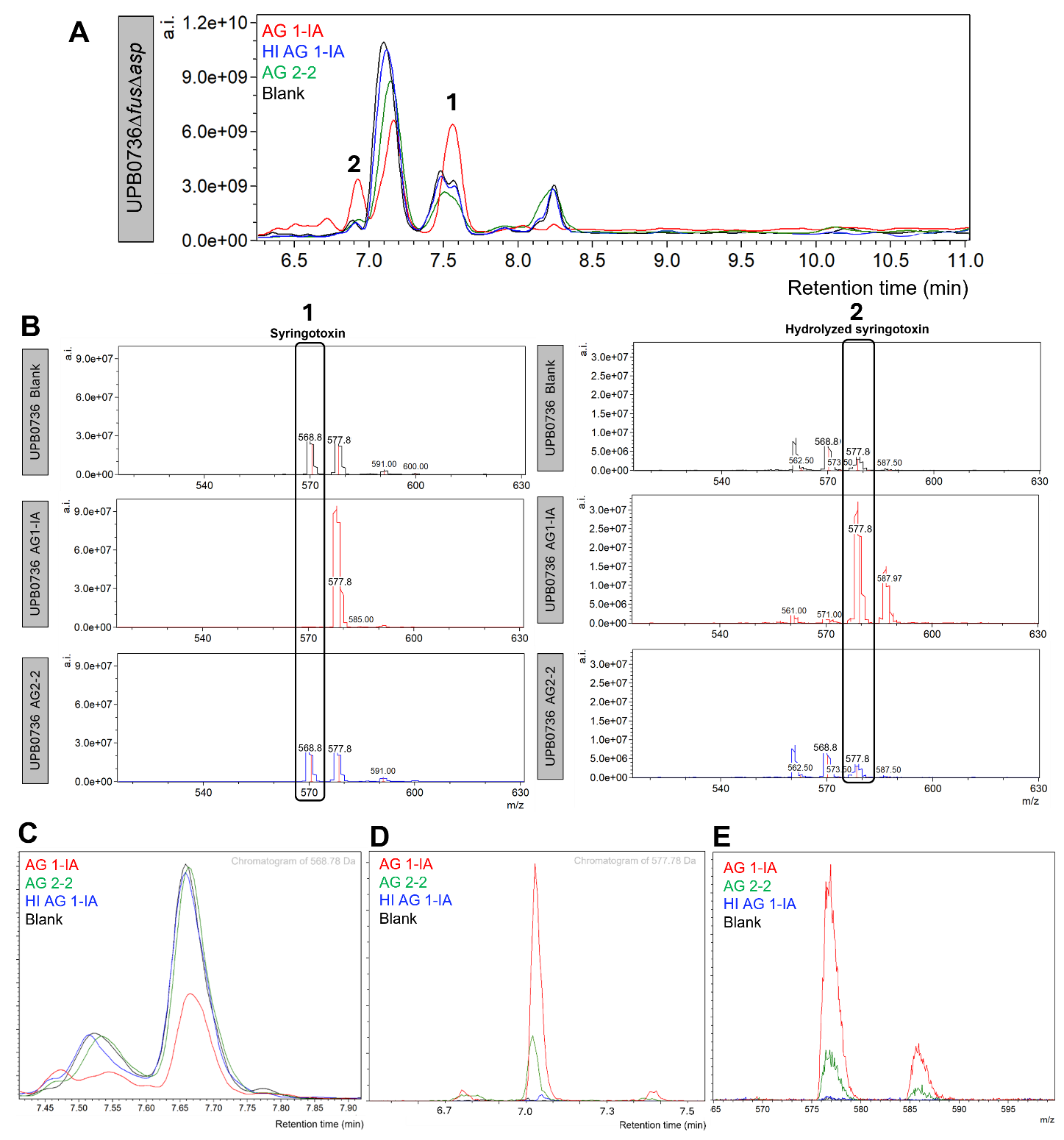


**Figure S4.** (**A**) UPLC-MS scan (cone 30V) and spectra of *P. fuscovaginae* UPB0736 *∆fus∆asp* supernatant (SN) combined with the enzymes of AG 1-IA, heat inactivated (HI) AG 1-IA, AG 2-2 and a blank. (**B**) MS spectra of syringotoxin (mass-to-charge ratio (m/z) 568.8 [M+2H]^2+^) and hydrolyzed syringotoxin (m/z 577.8 [M+2H]^2+^) in the treatments.


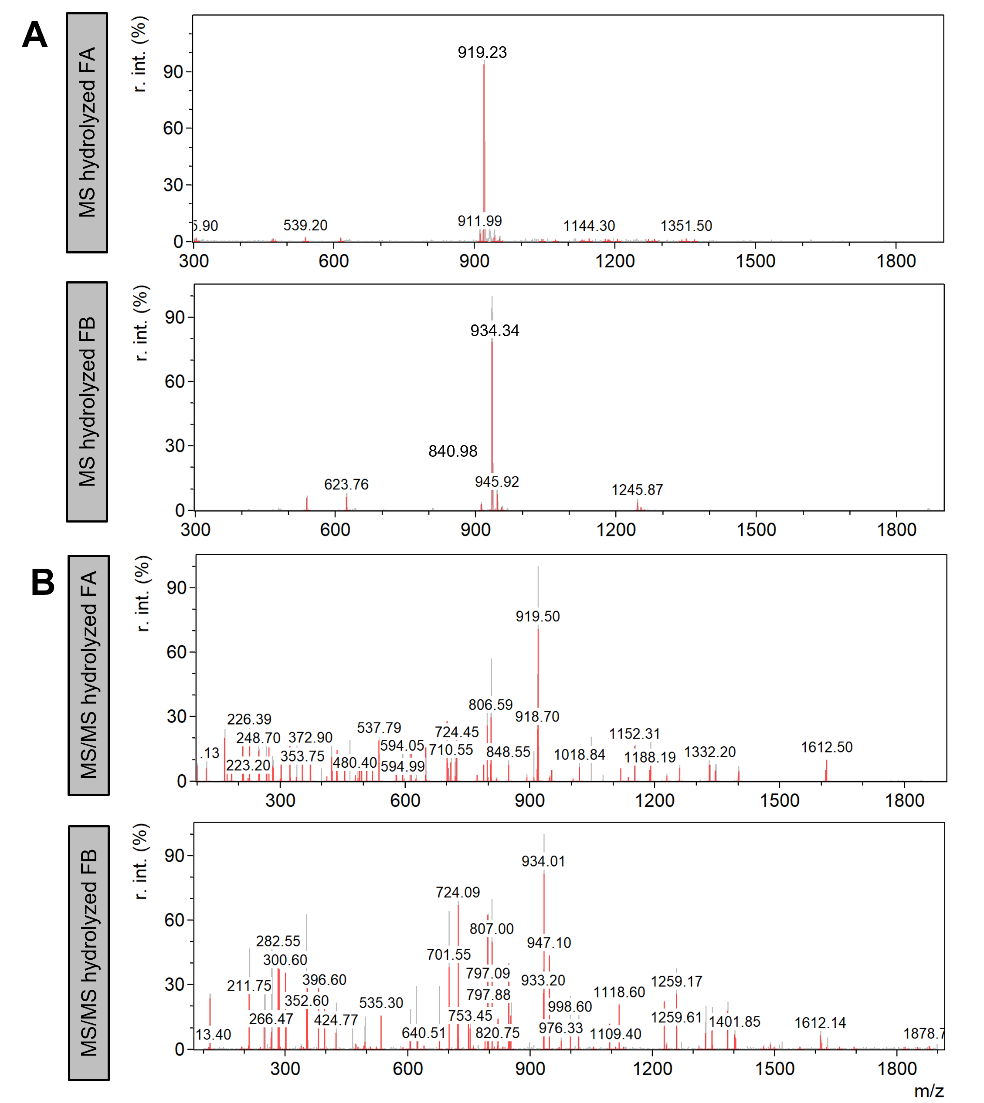


**Figure S5.** (**A**) The MS (cone 30V) and (**B**) MS/MS spectrum (cone 30V, CE 25V) of chemically (NaOH) hydrolyzed fuscopeptin A (FA, m/z 919.02 [M+2H]^2+^) and hydrolyzed fuscopeptin B (FB, m/z 933.04 [M+2H]^2+^).


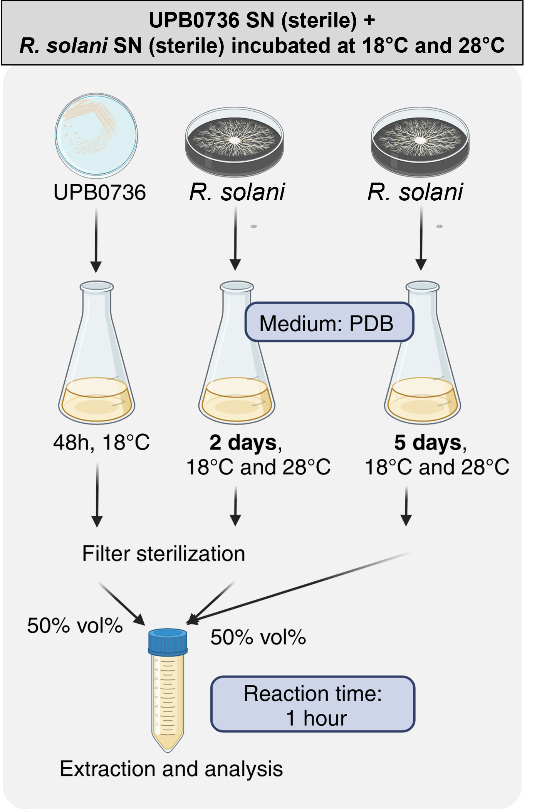


**Figure S6.** Methodology for the determination of CLiP degradation (produced by *P. fuscovaginae* UPB0736). The assay was conducted in the presence of *R. solani* supernatant derived from potato dextrose broth inoculated with *R. solani* plugs and incubated for 2 or 5 days at either 18°C or 28°C.


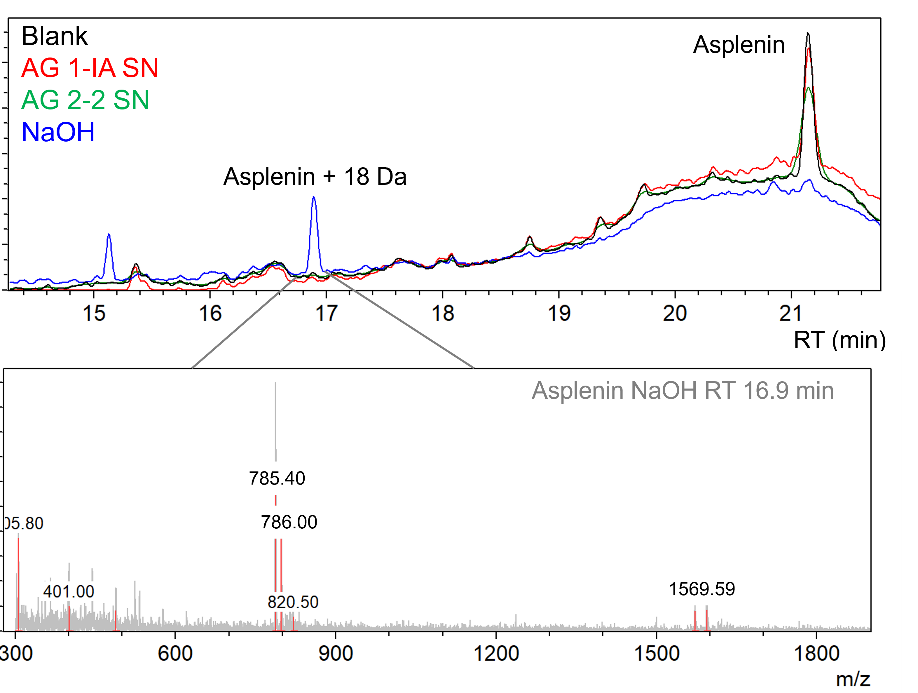


**Figure S7.** UPLC-MS scan (cone 30V) was performed on asplenin produced by the *P. fuscovaginae* mutant strain UPB0736*∆fst∆fus*, which lacks syringotoxin and fuscopeptin production. Intact asplenin (m/z 1551.9 [M+H]^+^), and the double charged ion (m/z 776.45 [M+2H]^2+^) was detected at a retention time of 21.1 min. The filter-sterilized supernatant (SN) containing asplenin was combined with *R. solani* AG 1-IA or AG 2-2 SN and, separately, subjected to alkaline hydrolysis using NaOH. Hydrolyzed asplenin (m/z 1569.9 [M+H]^+^) and its double charged ion (m/z 785.45 [M+2H]^2+^) were detected only in the NaOH-treated sample at a retention time of 16.9 min.


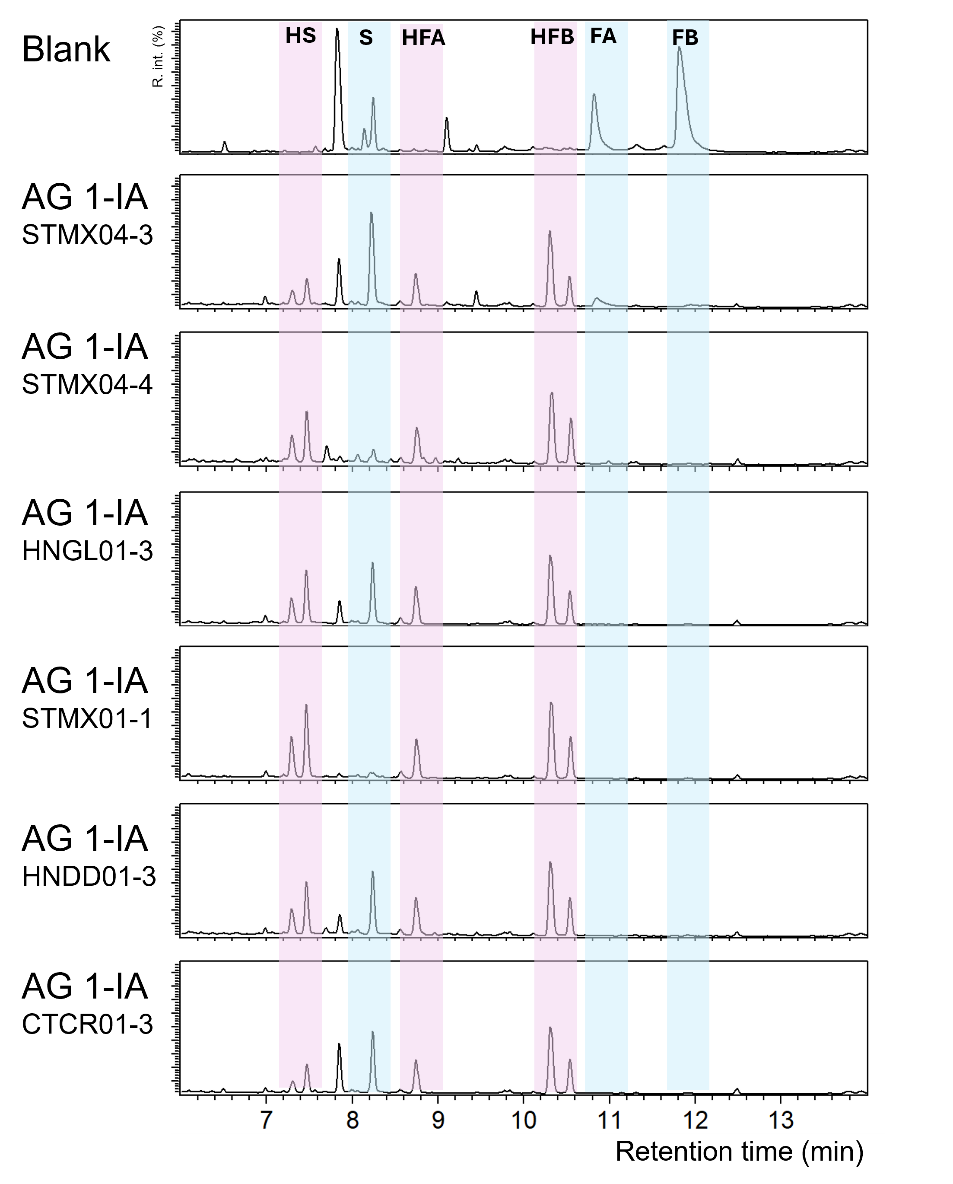


**Figure S8.** Full MS scan (cone 30V) of *P. fuscovaginae* UPB0736 supernatant (SN) mixed with the SN of different *R. solani* isolates from AG 1-IA. S: syringotoxin, HS: hydrolyzed syringotoxin, FA: fuscopeptin A, HFA: hydrolyzed fuscopeptin A, FB: fuscopeptin B, HFB: hydrolyzed fuscopeptin B.


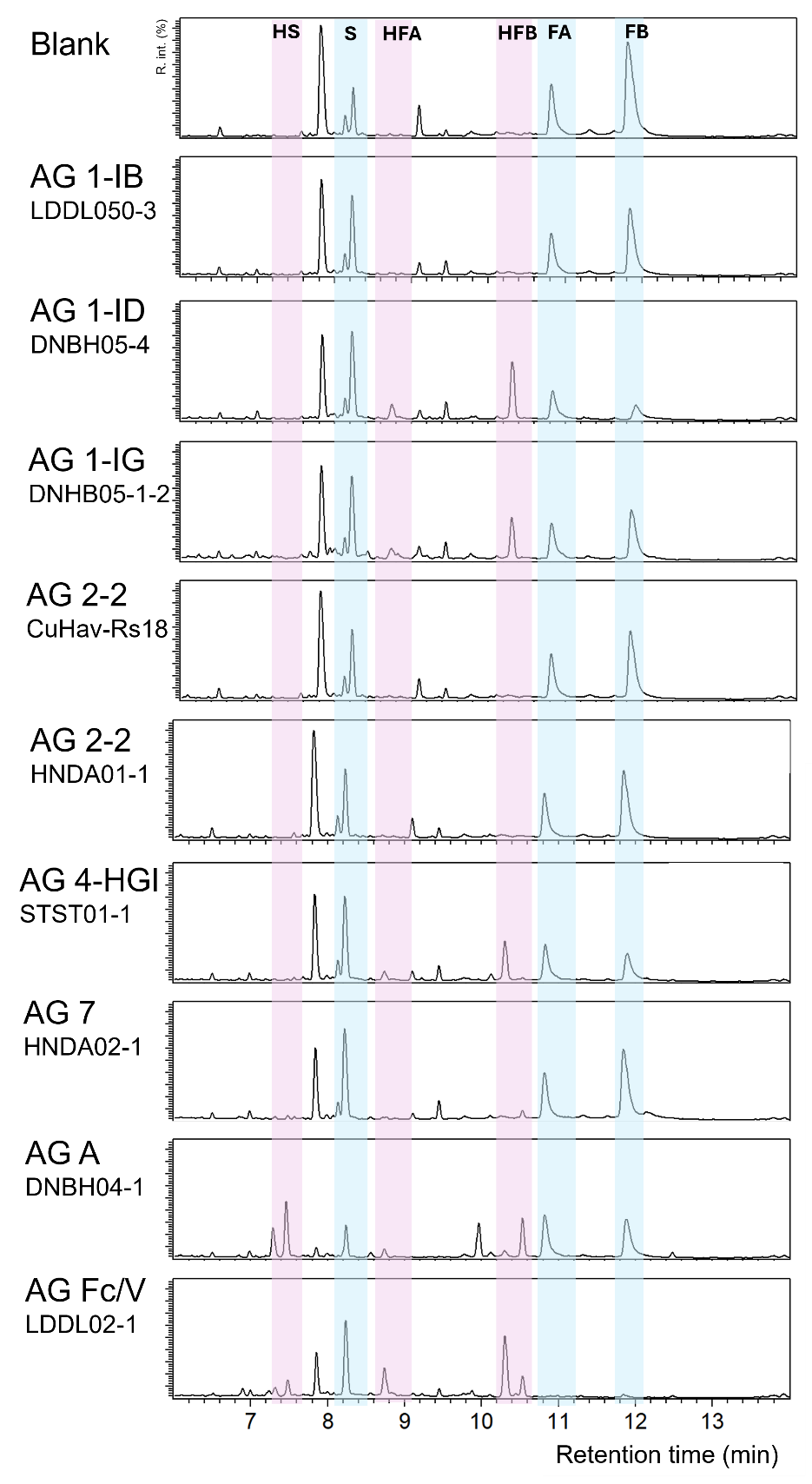


**Figure S9.** Full MS scan (cone 30V) of *P. fuscovaginae* UPB0736 supernatant (SN) mixed with the SN of different *R. solani* isolates. S: syringotoxin, HS: hydrolyzed syringotoxin, FA: fuscopeptin A, HFA: hydrolyzed fuscopeptin A, FB: fuscopeptin B, HFB: hydrolyzed fuscopeptin B.


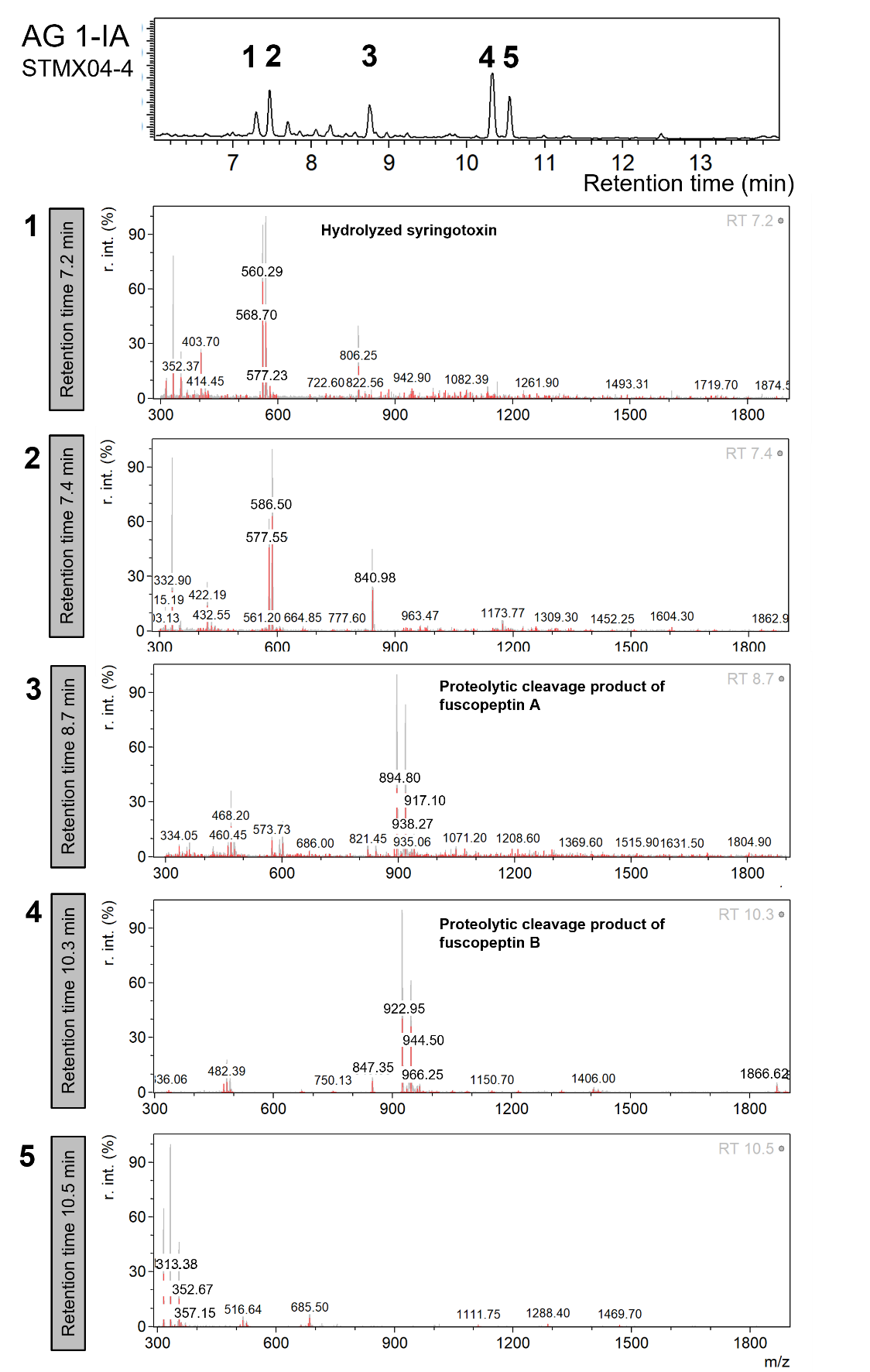


**Figure S10.** MS spectra (cone 30V) of the peaks occurring at the chromatogram of *P. fuscovaginae* UPB0736 supernatant (SN) mixed with the SN of *R. solani* AG 1-IA STMX04-4, (1) corresponds to hydrolyzed syringotoxin, (3) and (4) correspond to the products of protease-mediated cleavage at the glycine-alanine peptide bond in fuscopeptin A and fuscopeptin B, respectively and (5) corresponds to an extra peak that is likely linked to syringotoxin degradation.

**Table S1.** MS/MS parameters. Listed are the retention times (RT), parent ions and their corresponding daughter ions, as well as the cone voltage and collision energy (CE) applied.

| Family | Component | RT (min) | Fragment identity (m/z) | | Daughter  Fragments | Cone (V) | CE (V) |
| --- | --- | --- | --- | --- | --- | --- | --- |
| **Mycin** | Syringotoxin | 7.6 | **1136.56**  568.78  1158.54 | **[M+H]^+^**  [M+2H]^2+^  [M+Na]^+^ | 1118.8  741.19  723.1  374.1  559.94  467.88  374.1 | 60  60  60  60  30  30  30  60 | 50  50  25  50  20  25  25 |
| **Peptin** | Fuscopeptin A | 9.7 | **1819.04**  910.02  1841.02 | **[M+H]^+^**  [M+2H]^2+^  [M+Na]^+^ | **797.70**  1313.00  1383.70  1001.30 | 60  30  30  30  30  60 | 30  30  30  30 |
|  | Fuscopeptin B | 10.7 | **1847.08**  924.04  1869.06 | **[M+H]^+^**  [M+2H]^2+^  [M+Na]^+^ | **797.70**  1312.70  1384.10  1001.30  1592.90 | 60  30  30  30  30  30  60 | 30  30  30  30  30 |
| **Asplenin** | Asplenin | 19.9 | **1551.95**  776.45  1573.9 | **[M+H]^+^**  [M+2H]^2+^  [M+Na]^+^ | 1161.8  1268.82  625.57 | 40  40  20  40 | 30  30  15 |

**Table S2.** Measured syringotoxin concentrations (mg/L) in broth containing *P. fuscovaginae* UPB0736, for different treatments. Significances calculated using the pairwise Wilcoxon rank sum test (p<0.05, n=3). Numbers with the same letters are not significantly different from each other.

| Test | Treatments | UPB0736 | |
| --- | --- | --- | --- |
|  |  | mg/L | SD |
| Supernatant | Blank1 | 2.59 a | 0.86 |
|  | *R. solani* AG 1-IA STMX04-3 | 0.35 c | 0.25 |
|  | *R. solani* AG 2-2 CuHav-Rs18 | 0.24 c | 0.04 |
| Plugs | Blank2 | 4.01 a | 0.48 |
|  | *R. solani* AG 1-IA STMX04-3 plug | 1.50 b | 0.42 |
|  | *R. solani* AG 2-2 CuHav-Rs18 plug | 1.25 b | 0.32 |

**Table S3.** Concentrations of syringotoxin, fuscopeptin A and B in mg/L when mixed with the supernatant of different isolates of *R. solani*.


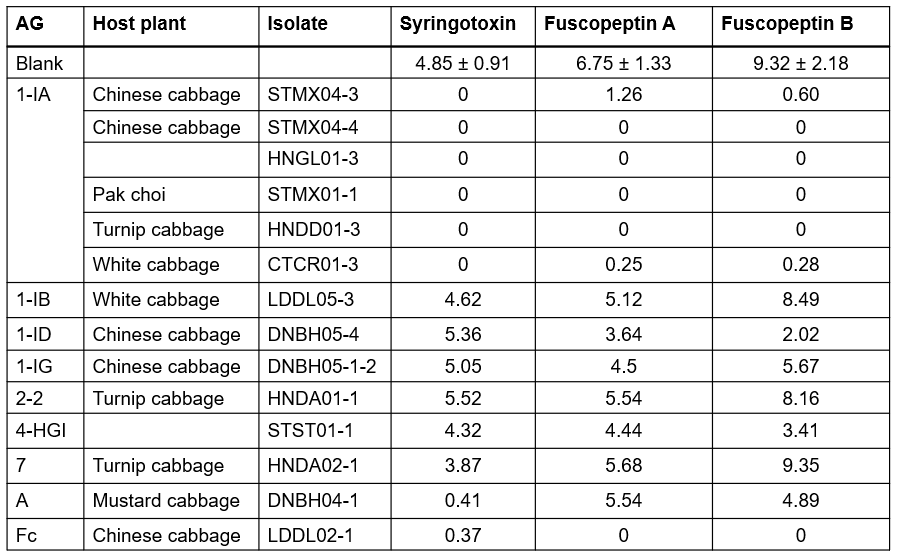


**Table S4.** pH measurements of the *R. solani* AG supernatants (potato dextrose broth)

| *R. solani* AG | Isolate | pH |
| --- | --- | --- |
| Blank | / | 5.21 |
| AG 1-IA | STMX04-3 | 7.45 |
| AG 1-IA | STMX04-4 | 7.00 |
| AG 1-IA | HNGL01-3 | 5.88 |
| AG 1-IA | STMX01-1 | 6.59 |
| AG 1-IA | HNDD01-3 | 6.49 |
| AG 1-IA | CTCR01-3 | 6.28 |
| AG 1-IB | LDDL050-3 | 6.40 |
| AG 1-ID | DNBH05-4 | 4.76 |
| AG 1-IG | DNB05-1-2 | 5.96 |
| AG 2-2 | CuHav-Rs18 | 5.37 |
| AG 2-2 | HNDA01-1 | 5.58 |
| AG 4-HGI | STST01-1 | 6.01 |
| AG 7 | HNDA02-1 | 4.69 |
| AG A | DNBH04-1 | 6.34 |
| AG Fc/V | LDDL02-1 | 4.52 |
